# Associations between Depression, Anxiety, Fatigue, and Learning Motivating Factors in e-Learning-Based Computer Programming Education

**DOI:** 10.3390/ijerph18179158

**Published:** 2021-08-30

**Authors:** Aiste Dirzyte, Aivaras Vijaikis, Aidas Perminas, Romualda Rimasiute-Knabikiene

**Affiliations:** 1 Faculty of Creative Industries, Vilnius Gediminas Technical University, 10221 Vilnius, Lithuania; 2 Institute of Psychology, Mykolas Romeris University, 08303 Vilnius, Lithuania; aivarasvijaikis@gmail.com (A.V.); romualda.rimasiute@gmail.com (R.R.-K.); 3 Department of Psychology, Vytautas Magnus University, 44248 Kaunas, Lithuania; aidas.perminas@vdu.lt

**Keywords:** depression, anxiety, fatigue, learning, motivating factors

## Abstract

Quarantines imposed due to COVID-19 have forced the rapid implementation of e-learning, but also increased the rates of anxiety, depression, and fatigue, which relate to dramatically diminished e-learning motivation. Thus, it was deemed significant to identify e-learning motivating factors related to mental health. Furthermore, because computer programming skills are among the core competencies that professionals are expected to possess in the era of rapid technology development, it was also considered important to identify the factors relating to computer programming learning. Thus, this study applied the Learning Motivating Factors Questionnaire, the Patient Health Questionnaire-9 (PHQ-9), the Generalized Anxiety Disorder Scale-7 (GAD-7), and the Multidimensional Fatigue Inventory-20 (MFI-20) instruments. The sample consisted of 444 e-learners, including 189 computer programming e-learners. The results revealed that higher scores of individual attitude and expectation, challenging goals, clear direction, social pressure, and competition significantly varied across depression categories. The scores of challenging goals, and social pressure and competition, significantly varied across anxiety categories. The scores of individual attitude and expectation, challenging goals, and social pressure and competition significantly varied across general fatigue categories. In the group of computer programming e-learners: challenging goals predicted decreased anxiety; clear direction and challenging goals predicted decreased depression; individual attitude and expectation predicted diminished general fatigue; and challenging goals and punishment predicted diminished mental fatigue. Challenging goals statistically significantly predicted lower mental fatigue, and mental fatigue statistically significantly predicted depression and anxiety in both sample groups.

## 1. Introduction

Computer programming skills and computational literacy are among the core competencies that professionals are expected to possess in the era of rapid technology development and a fast-changing work environment [1]. 

Research indicates that learners of various ages are motivated to acquire computer programming skills because computer programming generates cognitive benefits [2] and may ensure more extensive possibilities of work or entrepreneurship [3]. For example, Python is a general-purpose programming language that empowers developers to use several different programming styles when creating programs. According to HackerRank, Python is the second-most in-demand programming language, and over 50% of hiring managers seek candidates who know this language [4]. The programming languages of Python, Java, HTML, CSS, SQL, NoSQL, C#, Rust, Perl, and Go are the most demanded in 2021, and courses aimed at learning these programming languages are in high demand [5].

However, computer programing is one of the most challenging learning tasks [6]. The rates of dropout or failure in programming language courses are higher than those of other disciplines [7]. Various studies suggest that the problem of programming learning failures is related to “subject difficulty” [7], course design and the learning context [8], lack of collaborative work [9], learners’ characteristics [10], and learning motivation [11,12].

### 1.1. Learning Motivating Factors

Research indicates that learning motivation is a crucial factor in determining learning outcomes [11,13,14], because motivated learners make more effort, and are more attentive and more persistent in the face of difficulties [15]. Motivation in education can be context specific, and motivation also relates to the level of engagement with a topic and the nature of that involvement [16]. Studies suggest that learning motivation depends on students’ attitudes and expectations, reward and recognition, challenging goals, social pressure, and competition [7]. 

In 2010, Law et al. [6] proposed a model of combination of intrinsic and extrinsic learning motivation factors: (1) an individual attitude and expectation factor, based on expectancy, instrumentality, and valence; (2) a challenging goals factor, based on the assumption that personal goals are essential in determining performance; (3) a clear direction factor, based on research indicating that effective learning is associated with the learner’s perception of a clear direction; (4) a reward and recognition factor, based on reinforcement theory, suggesting that proper reward and recognition can be a crucial motivator of learning; (5) a punishment factor, based on the assumption that punishments, and the expectation of punishments, motivate some people, yet may also act as a demotivating factor; (6) a social pressure and competition factor, based on evidence that social forces such as peer pressure and competition affect learning; and (7) an efficacy factor, based on the assumption that learning efficacy or learning-related self-efficacy refers to what a person believes he or she can do in a particular learning task. In this model, intrinsic motivation encompasses motivational factors of individual attitude and expectation, and challenging goals, whereas extrinsic motivation includes clear direction, reward and recognition, punishment, social pressure, and competition [6].

The theoretical model of Law et al. [6] on learning motivating factors is based on several eminent theories and previous research. Expectancy theory [17] suggests that motivation is a multiplicative function of expectancy (people have different expectations and levels of confidence in their capabilities), instrumentality (the perceptions of whether it is possible to obtain the desired outcome), and valence (the emotional orientations regarding outcomes or rewards), which provides the basis for the learning motivating factor of individual attitude and expectation. Furthermore, several studies reported the significance of personal goals in performance [18,19,20], which provides the basis for the learning motivating factor of personal goals. In addition, several studies have demonstrated that learners respond more positively when given a clear direction [21,22], which provides the basis for the learning motivating factor of clear direction. Furthermore, reinforcement theory implies that the anticipation of performance evaluation can affect the motivational direction and task involvement during task performance. These motivational processes may influence subsequent interest in the task, because proper reward and recognition can motivate learning [23]. These findings provide the basis for the learning motivating factor of reward and recognition. Reinforcement theory also implies that rewards and expectation of rewards, and punishments and expectation of punishments, can motivate some people. However, excessive punishment can be demotivating [24]. These results provide the basis for the learning motivating factor of punishment. Next, Law et al. [6] noted that “peer-learning among students in higher education is increasingly a meaningful and important topic for research” [6]. Several studies have demonstrated that peer pressure and competition can also influence learning, and this influence can be both harmful and beneficial [25,26,27], which provides the basis for the learning motivating factor of social pressure and competition. Furthermore, several studies have reported that learning-related self-efficacy is related to academic performance [28,29,30,31], which provides the basis for the learning motivating factor of efficacy. 

Although numerous authors analyzed learning motivation in computer programming learning, some confusing findings [3,32,33] indicate that computer learners’ learning motivating factors are under-researched.

### 1.2. Learning and Emotional Health

Research indicates that learning motivation can be impacted by numerous factors, including emotional health, which leads to success in work, relationships, and learning [19]. However, learning motivation, which is “about the nature of that involvement” [16] (p. 111), may also affect emotional health, resulting in anxiety, depression, and emotional exhaustion. 

Recent research suggests that emotions play an essential role in the ability of students to master complex intellectual activities such as computer programing. Moreover, emotions are essential factors in determining whether the student will master the exercise of learning to program in the short and long terms [34]. Some studies suggest that intrinsic learning motivation is related to better learning outcomes and higher satisfaction with the learning process, whereas extrinsic motivation is related to higher stress levels [35,36]. 

Several affective states that students experience when learning to program (e.g., boredom) were found to be related to performance, whereas the impact of certain affective states (e.g., anxiety or sadness) was not demonstrated [37,38,39]. Confusion, frustration, and boredom were found to be the most frequent affective states negatively correlated with performance when learning to program computers [39]. Prolonged confusion was found to harm learning outcomes, but the negative effect of boredom was even more substantial [40]. However, some research suggests that anxiety, happiness, anger, surprise, disgust, sadness, and fear are uncommon affective states when learning to program, and are unrelated to learning outcomes [38,39]. 

Studies analyzing the role of emotions in computer programming learning have focused on self-reported emotions. Research has analyzed students’ affective states, captured in a natural learning environment [41]. Studies have analyzed emotions, captured by web cameras and microphones when students were accessing the e-learning environments [42] or used semantic analysis (sentiment analysis), facial expression, and physiological signals [43], analyzed dialogues, body language, and facial expressions [44], and applied bimodal audiovisual emotion recognition [45]. 

However, although the question “How does the learner feel?” has received a significant amount of attention in computer programming e-learning research, the questions “How has the learner been feeling lately?” and “How are the learner’s recent affective states related to learning motivation”? are under-researched. It is unclear whether the prolonged affective states impact learning motivation and how different affective states are related to specific learning motivating factors. 

Based on previous studies, it can be presumed that intrinsic motivation may enhance states of flow or zest, which are the opposites to depression, anxiety, or fatigue [46,47], whereas extrinsic motivation, especially fear of punishment, may lead to intensified anxiety [48,49,50]. The increase in anxiety levels for extended periods may result in depression, accompanied by mental or physical fatigue. However, the links between learning and anxiety, depression, and fatigue need thorough investigation.

### 1.3. Anxiety, Depression, and Fatigue

Anxiety is one of the primary emotions and a multidimensional reaction to actual or potential dangers, which combines somatic, cognitive, emotional, and behavioral components representing evolutionary mechanisms to survive or cope with the threatening stimuli. However, the response may become excessive or maladaptive under certain circumstances and manifest in anxiety disorders [51]. 

From the neurobiological perspective, human anxiety reactions are assumed to be mediated by the genetic background; the amygdala, hippocampus, cingulate cortex, hypothalamus, serotonergic, gamma-aminobutyric acidergic, and adrenocortical systems; and various brainstem areas [51,52]. Furthermore, the nature–nurture interplay is viewed as possibly increasing the risk of developing excessive anxiety; for example, in the brain-derived neurotrophic factor gene (BDNFMet at codon 66) in interaction with early life, stress was found to predict neuroticism and higher anxiety [53]. However, the cognitive theory presumes that anxiety reactions are predominantly cognitive phenomena [54,55,56]. The anxiety cycle can be significantly influenced by triggering events or stimuli and predisposing factors (e.g., trait anxiety) [55,56].

According to the tripartite model of anxiety and depression [57], the specific characteristic of anxiety is physiological hyperarousal, which includes tension, nervousness, shakiness, and panic symptoms, and the specific characteristic of depression is anhedonia, the absence of positive affect, and dysthymia, the depressed mood [58]. Furthermore, research suggests a dual interaction between depression and fatigue, in which one increases the risk of the other [59].

### 1.4. Mental Health and e-Learning Education

Several studies have demonstrated the relationship between neuroticism, which reflects trait anxiety and depression [57], and learning achievements [60], evidencing that anxiety and depression lead to diminished performance. Research has also revealed that neuroticism is positively related to extrinsic academic motivation [61]. However, the importance of mental health in e-learning education is under-researched, despite attempts to conceptualize challenges in e-learning education [62,63,64].

Identifying challenges in e-learning education is especially significant because, as a result of the coronavirus outbreak, numerous countries face changes in many sectors, including education. Many students globally have been affected by the suspension of classes, and countries have faced the need for rapid implementation of e-learning. As a result, the question of learning motivation in e-learning is extremely important, particularly considering the observed statistically significant increase in the rates of anxiety and depression [65] and chronic fatigue [66].

Research suggests that university students were susceptible to developing depression and anxiety during the COVID-19 quarantine due to the psychologically challenging circumstances they faced each day [67,68]. Although anxiety levels may depend on coping strategies [69], and depression levels may depend on social support [70], social isolation during the COVID-19 pandemic can intensify feelings of worthlessness, and presumed dangers can increase worrying and anxiety, which combined may finally lead to depression and emotional exhaustion [71]. 

Several studies revealed that most students during the quarantine experienced increased anxiety and depression [70,72,73]. Furthermore, research found a significant relationship between students’ satisfaction with e-learning and the prevalence of depression and anxiety symptoms [67]. Shockingly, during the COVID-19 pandemic, more than half of students met the diagnostic criteria of a generalized anxiety disorder (52%) and depression (63%) [74]. Moreover, although some mild anxiety was related to an increase in motivation [75], the states of severe anxiety and depression were related to dramatically diminished e-learning motivation, or even its absence [76]. 

To summarize, associations between learning motivating factors and depression, anxiety, and fatigue are under-researched, but there is some evidence that these states may relate to constrained learning motivation. From the perspective of cognitive processes, one of the significant factors contributing to the inhibition of learning motivation is subjective fatigue [77,78], which refers to a decline in mental efficiency and the accompanying feelings of weariness [79,80], and more generally represents a crucial element in the effort-regulation system as a whole [81]. By comparison, extrinsic motivation may lead to emotional exhaustion, and mental and general fatigue, but the paths and contributing factors are under-researched. 

Therefore, it is important to analyze the associations between the learning motivating factors and depression, anxiety, and fatigue in e-learning-based education. Furthermore, to identify the specific factors relating to computer programming e-learning, it is essential to analyze the differences between those who participate in e-learning-based computer learning courses and those who study in other university programs. Consequently, although this study targeted students enrolled in e-learning-based computer programming education, to reveal the specifics of programming e-learners, the comparative group consisted of university students who studied social sciences. Due to the COVID-19 pandemic, these students were studying remotely. 

Thus, this study aimed to identify associations between depression, anxiety, fatigue, and learning motivating factors in e-learning-based education. Furthermore, this study aimed to compare the patterns in participants and non-participants of e-learning-based computer programming courses, and to identify the specific factors relating to computer programming e-learners. 

Based on the previous research, we hypothesized that:

**Hypotheses** **1** **(H1).** *Lower scores of depression relate to higher scores of learning motivating factors;*

**Hypotheses** **2** **(H2).** *Lower anxiety scores relate to higher scores of intrinsic, but not extrinsic, learning motivating factors;*

**Hypotheses** **3** **(H3).** *Lower scores of general fatigue relate to higher scores of learning motivating factors;*

**Hypotheses** **4** **(H4).** *Extrinsic learning motivating factors predict increased anxiety, and intrinsic learning motivating factors predict decreased anxiety;*

**Hypotheses** **5** **(H5).** *Extrinsic learning motivating factors predict increased depression, and intrinsic learning motivating factors predict decreased depression;*

**Hypotheses** **6** **(H6).** *Extrinsic learning motivating factors, anxiety, and depression predict increased general and mental fatigue, and intrinsic learning motivating factors predict decreased general and mental fatigue;*

**Hypotheses** **7** **(H7).** *Associations exist between learning motivating factors, anxiety, depression, and fatigue, but they differ between participants and non-participants of e-learning-based computer programming courses.*

## 2. Materials and Methods

### 2.1. Sample

In the full sample of 465 participants, a total of 444 participants had no missing data. Because the number of cases with missing values was small, we used listwise deletion of cases with missing values. Therefore, all analyses were conducted using a sample of 444 individuals. The sample’s breakdown by gender was 32.7 percent male (*n* = 145) and 67.3 percent female (*n* = 299). The respondents’ mean age was 25.19 years (standard deviation (*SD)* = 8.268, 95% confidence interval (CI) = 24.42, 25.97, age range = 18–57 years). A total of 189 (42.6%) of participants studied in e-learning-based computer programming courses organized by Turing College. Based on Ding, Velicer, and Harlow [82], a sample size within the range of 100 to 200 is sufficient for structural equation modeling [82]. Thus, the sample size of the computer programming e-learners in this study was satisfactory to obtain meaningful results in the applied statistical models.

The comparative group consisted of 255 (57.4%) respondents who studied social sciences at various Lithuanian Universities; however, these students were studying remotely due to the COVID-19 pandemic. 

At the time of the research, both groups of e-learners (computer programming and social sciences) were undertaking their studies. E-learners were informed about the study by e-mail and provided their consent to participate in the research. Participation in the study was voluntary, and the participants did not receive any compensation. The procedure was administered online at https://www.psytest.online (accessed on 7 July 2021) and followed the General Data Protection Regulation (GDPR) guidelines. The study was approved by the Institutional Review Board of the Institute of Management and Psychology, based on the approval of the Biomedical Research Scientific-Ethics Committee at Klaipėda University (No. STIMC—BMTEK-P03, 12 April 2021). 

### 2.2. Instruments

This study applied four instruments: the translated Lithuanian version of the Learning Motivating Factors Questionnaire [6,83], the translated Lithuanian version of the Patient Health Questionnaire-9 (PHQ-9) [84], the translated Lithuanian version of the Generalized Anxiety Disorder Scale (GAD-7) [85], and the translated Lithuanian version of the Multidimensional Fatigue Inventory (MFI-20) [86]. To ensure that the Lithuanian items corresponded as closely as possible to the English items, the original items of both instruments were translated into Lithuanian and back-translated.

#### 2.2.1. The PHQ-9

To assess depression, we applied the Patient Health Questionnaire-9 (PHQ-9) [84], which is a 9 item self-reported measure used to assess depression severity and criteria for a major depressive episode (MDE). Items are assessed for symptoms of depression (e.g., “little interest or pleasure in doing things”), and response anchors range temporally from 0 (not at all) to 3 (nearly every day). Items are summed to create a severity score ranging from 0 to 27, with higher scores reflecting greater depression severity. Scores above 10 are considered to be in the depressive area [84]. The scores of the PHQ-9 are usually used as categorical variables due to their clinical relevance. In this study, we present only frequencies and ANOVA results based on the PHQ-9 categories. Due to the relatively small sample size, we used the PHQ-9 as a continuous variable to apply the SEM, because research has suggested that the PHQ-9 can also be examined as a continuous variable [84]. To apply the SEM with categorical variables, a considerably larger sample in each category is needed. Unfortunately, the number of participants in each category was insufficient for the chosen statistical models.

Several validation studies confirmed the one-dimensional structure of the PHQ-9, evidencing the instrument’s internal consistency, with α = 0.87 [84,87].

#### 2.2.2. The GAD-7

To assess anxiety, we applied the Generalized Anxiety Disorder Scale (GAD-7) [85], which is a 7 item self-reported questionnaire that assesses symptoms of general anxiety according to a 4 point Likert-type scale, ranging from 0 (not at all) to 3 (nearly every day). These seven items assess (1) feeling nervous, anxious, or on edge; (2) being unable to stop or control worrying; (3) worrying too much about different things; (4) having trouble relaxing; (5) being restless; (6) becoming easily annoyed or irritable; and (7) feeling afraid as if something awful might happen. Items are summed to create a severity score ranging from 0 to 21. Scores above 10 are considered to be in the clinical range [85]. The scores of the GAD-7 are usually used as categorical variables due to their clinical relevance. In this study, we present only frequencies and ANOVA results based on the GAD-7 categories. Due to the relatively small sample size, we used the GAD-7 as a continuous variable to apply the SEM, because research has suggested that the GAD-7 can also be examined as a continuous variable [85]. To apply the SEM with categorical variables, a considerably larger sample in each category is needed. Unfortunately, the number of participants in each category was insufficient for the chosen statistical models.

Based on a validation study in the general population, the CFA results substantiated the one-dimensional structure of the GAD-7 and its factorial invariance for gender and age [88]. Research reported that the internal consistency α of the GAD-7 was 0.89, and intercorrelations with the PHQ-2 and the Rosenberg Self-Esteem Scale were *r* = 0.64 (*p* < 0.001) and *r* = −0.43 (*p* < 0.001) [88].

#### 2.2.3. The MFI-20

To assess fatigue, we applied the Multidimensional Fatigue Inventory (MFI-20) [86]. The MFI-20 consists of 20 items categorized to five dimensions: general fatigue, physical fatigue, reduced activity, reduced motivation, and mental fatigue. For each scale, two items are oriented in the direction of fatigue, whereas the other two items are oriented in the opposite direction. Example items for the scale “general fatigue” are “I feel tired” and “I feel fit.” The responses to each item are captured with a five-point Likert scale, ranging from 1 (yes, this is true) to 5 (no, this is not true). MFI-20 convergent validity was verified by correlating the hospital anxiety and depression scale (HADS) and the global quality of life scale of the EORTC QLQ-C30. Research has reported that the internal consistency α of the MFI-20 instrument was 0.72–0.87 [89].

#### 2.2.4. The Learning Motivating Factors Questionnaire

To assess learning motivation, we applied the Learning Motivating Factors Questionnaire, developed by Law et al. [6,83]. This 19 item questionnaire measures factors that have a positive motivating effect on learning and covers several motivational variables listed below. The individual attitude and expectation subscale measures the student’s attitude and expectation towards learning. The challenging goals subscale measures perceived challenging goals in learning. The clear direction subscale measures perceived specified direction in learning. The reward and recognition subscale measures perceived positive reinforcements, such as reward, appreciation, and encouragement. The punishment subscale measures the perceived negative reinforcement due to punishment. The social pressure and competition subscale measures perceived forces of pressure and competition from peers. The e-effect subscale measures the perceived effect of the e-learning setting. The response pattern followed a 6-point Likert scale, ranging from 1 (disagree very much) to 6 (agree very much). The validity of the construct was verified through oblique rotation exploratory factorial analysis. Research reported that the internal consistency α of the instrument was 0.95. The discriminant validity of each construct was checked using a multi-trait matrix [6].

In this study, for reliability analysis, Cronbach’s alpha indexes were calculated. Cronbach alphas for the used instruments (the PHQ-9, the GAD-7, the MFI-20, the Learning Motivating Factors Questionnaire) in this research sample are presented in Table 1.

### 2.3. Statistical Analysis

For data analysis, we used SPSS v.26.0 (IBM Corp., Armonk, NY, USA). The structural equation modeling (SEM), confirmatory factor analysis (CFA) models were conducted using AMOS v.26.0 (IBM Corp., Armonk, NY, USA) and JASP v. 0.14.1.0 (University of Amsterdam, Amsterdam, The Netherlands). Model fit was evaluated based on the CFI (Comparative Fit Index), the Normed Fit Index (NFI), the Tucker–Lewis coefficient (TLI), RMSEA (Root Mean Square Error of Approximation), and SRMR (Standardized Root Mean Square Residual), whereas the χ^2^ was used for descriptive purposes only because it is highly sensitive to sample size [90]. The values higher than 0.90 for CFI, NFI, and TLI, and values lower than 0.08 for RMSEA and SRMR, were considered as indicative of a good fit [91]. In this research, we considered *p*-values less than 0.05 to be statistically significant [92].

The Shapiro–Wilk test showed the departure from normality for the variables of individual attitude and expectation *W* (406) = 0.960, *p* < 0.001; challenging goals *W* (406) = 0.965, *p* < 0.001; clear direction *W* (406) = 0.936, *p* < 0.001; reward and recognition *W* (406) = 0.934, *p* < 0.001; punishment *W* (406) = 0.967, *p* < 0.001; social pressure and competition *W* (406) = 0.984, *p* < 0.001; anxiety *W* (393) = 0.932, *p* < 0.001; depression *W* (393) = 0.948, *p* < 0.001; physical fatigue *W* (444) = 0.972, *p* < 0.001; reduced activity *W* (444) = 0.982, *p* < 0.001; reduced motivation *W* (444) = 0.980, *p* < 0.001; mental fatigue *W* (444) = 0.979, *p* < 0.001; general fatigue *W* (444) = 0.983, *p* < 0.001.

Similarly, the Kolmogorov–Smirnov test showed that data were non-normally distributed for the variables of individual attitude and expectation *D* (406) = 0.113, *p* < 0.001; challenging goals *D* (406) = 0.094, *p* < 0.001; clear direction *D* (406) = 0.139, *p* <0.001; reward and recognition *D* (406) = 0.142, *p* < 0.001; punishment *D* (406) = 0.086, *p* < 0.001; social pressure and competition *D* (406) = 0.067, *p* < 0.001; anxiety *D* (393) = 0.135, *p* < 0.001; depression *D* (393) = 0.112, *p* < 0.001; physical fatigue *D* (444) = 0.088, *p* < 0.001; reduced activity *D* (444) = 0.073, *p* < 0.001; reduced motivation *D* (444) = 0.080, *p* < 0.001; mental fatigue *D* (444) = 0.098, *p* < 0.001; general fatigue *D* (444) = 0.076, *p* < 0.001.

The distribution was moderately skewed: individual attitude and expectation skewness = −0.494 (Standard Error (*SE*) 0.121), kurtosis = −0.028 (*SE* = 0.242); challenging goals skewness = −0.435 (*SE* = 0.121), kurtosis = −0.190 (*SE* = 0.242); clear direction skewness = −0.435 (*SE* = 0.121), kurtosis = −0.176 (*SE* = 0.242); reward and recognition skewness = −0.661 (*SE* = 0.121), kurtosis = −0.045 (*SE* = 0.242); punishment skewness = −0.038 (*SE* = 0.121), kurtosis = −0.642 (*SE* = 0.242); social pressure and competition skewness = −0.030 (*SE* = 0.121), kurtosis = −0.500 (*SE* = 0.242); anxiety skewness = 0.805 (*SE* = 0.117), kurtosis = −0.120 (*SE* = 0.233); depression skewness = 0.655 (*SE* = 0.117), kurtosis = −0.327 (*SE* = 0.245); physical fatigue skewness = 0.137 (*SE* = 0.116), kurtosis = −0.864 (*SE* = 0.231); reduced activity skewness = 0.021 (*SE* = 0.116), kurtosis = −0.712 (*SE* = 0.231); reduced motivation skewness = 0.324 (*SE* = 0.116), kurtosis = −0.135 (*SE* = 0.231); mental fatigue skewness = 0.292 (SE = 0.116), kurtosis = −0.387 (*SE* = 0.231); general fatigue skewness = −0.100 (*SE* = 0.116), kurtosis = −0.557 (*SE* = 0.231).

Therefore, we conducted a square root transformation (SQRT) of significantly negatively skewed variables to create normally distributed variables and conduct the CFA analyses.

## 3. Results

The frequencies of the self-reported depression categories in this sample are presented in Table 2.

The frequencies of the self-reported anxiety categories in this sample are presented in Table 3.

The frequencies of the self-reported general fatigue categories in this sample are presented in Table 4.

The means, standard deviations, and correlations between the MFI-20 subscales in this study are reported in Table 5.

The means, standard deviations, and correlations between the Learning Motivating Factors Questionnaire subscales in this study are reported in Table 6.

The means, standard deviations, and correlations between the PHQ-9 and the GAD-7 scales in this study are reported in Table 7.

To test hypothesis 1 (H1), assuming that lower scores of depression relate to higher scores of learning motivating factors, we compared the scores of learning motivating factors based on the PHQ-9 categories (Table 8).

A one-way ANOVA was conducted to compare the effect of depression (PHQ-9) on learning motivating factors. We analyzed learning motivating factors in five groups: minimal depression, mild depression, moderate depression, moderately severe depression, and severe depression. An analysis of variance revealed that the effect of depression on individual attitude and expectation was significant, *F* (4, 387) = 3.324, *p* = 0.011. A Tukey post hoc test revealed that the scores of individual attitude and expectation were significantly higher in the minimal depression group (4.859, ±0.783) in comparison to the moderately severe depression group (4.403, ±0.904) (*p* = 0.018).

An analysis of variance revealed that the effect of depression on challenging goals was significant, *F* (4, 387) = 14.261, *p* < 0.001. A Tukey post hoc test revealed that the scores of challenging goals were significantly higher in the minimal depression group (4.833, ±0.808) in comparison to all groups: mild depression (4.354, ±1.091) (*p* = 0.003), moderate depression (4.703, ±1.026) (*p* < 0.001), moderately severe depression (3.708, ±1.079) (*p* < 0.001), and severe depression (3.844, ±1.173) (*p* < 0.001). Additionally, the scores of challenging goals were significantly higher in the mild depression group (4.354, ±1.091) in comparison to the moderately severe depression group (3.708, ±1.079) (*p* = 0.002). 

An analysis of variance revealed that the effect of depression on clear direction was also significant, *F* (4, 387) = 6.884, *p* < 0.001. A Tukey post hoc test revealed that the scores of clear direction were significantly higher in the minimal depression group (5.172, ±0.737) in comparison to the moderately severe depression (4.612, ±0.681) (*p* < 0.001) or severe depression (4.583, ±1.057) (*p* = 0.001) groups. Additionally, the scores of clear direction were significantly higher in the mild depression group (5.015, ±0.719) in comparison to the moderately severe depression group (4.612, ±0.681) (*p* = 0.014) and the severe depression group (4.583, ±1.057) (*p* = 0.033).

An analysis of variance revealed that the effect of depression on social pressure and competition was significant, *F* (4, 387) = 2.657, *p* = 0.033. A Tukey post hoc test revealed that the scores of social pressure and competition were significantly higher in the mild depression group (3.604, ±1.176) in comparison to the moderately severe depression group (2.975, ±1.198) (*p* = 0.016).

However, there were no significant differences between the scores of reward and recognition in the depression groups *F* (4, 387) = 0.972, *p* = 0.423. In addition, there were no significant differences between the scores of punishment in the depression groups, *F* (4, 387) = 1.901, *p* = 0.110.

Furthermore, to test hypothesis 2 (H2), which presumed that lower anxiety scores relate to higher scores of intrinsic, but not extrinsic, learning motivating factors, we compared the scores of learning motivating factors based on the GAD-7 categories (Table 9).

A one-way ANOVA was conducted to compare the effect of anxiety (GAD-7) on learning motivating factors. We analyzed learning motivating factors in four groups: minimal anxiety, mild anxiety, moderate anxiety, and severe anxiety. An analysis of variance revealed that the effect of severe anxiety on challenging goals was significant, *F* (3, 401) = 8.345, *p* < 0.001. A Tukey post hoc test revealed that the scores of challenging goals were significantly higher in the minimal anxiety group (4.604, ±1.020) in comparison to the moderate anxiety group (4.155, ±0.946) (*p* = 0.018) and the severe anxiety group (3.782, ±1.154) (*p* < 0.001). Additionally, challenging goals were significantly higher in the mild anxiety group (4.329, ±1.112) in comparison to the severe anxiety group (3.782, ±1.154) (*p* = 0.011).

An analysis of variance revealed that the effect of severe anxiety on clear direction was significant, *F* (3, 401) = 3.449, *p* = 0.017. Similarly, the effect of severe anxiety on social pressure and competition was significant, *F* (3, 401) = 2.784, *p* = 0.041. A Tukey post hoc test revealed that the scores of social pressure and competition were significantly higher in the minimal anxiety group (3.525, ±1.127) in comparison to the severe anxiety group (3.010, ±1.187) (*p* = 0.047). Additionally, scores of social pressure and competition were significantly higher in the mild anxiety group (3.564 ± 1.327) in comparison to the severe anxiety group (3.010, ±1.187) (*p* = 0.030).

However, there were no significant differences between the scores of individual attitude and expectation *F* (3, 401) = 1.234, *p* = 0.297, reward and recognition *F* (3, 401) = 1.466, *p* = 0.223, or punishment, *F* (3, 401) = 0.744, *p* = 0.526, in different anxiety groups. 

Next, to test hypothesis 3 (H3), assuming that lower scores of general fatigue relate to higher scores of learning motivating factors, we compared the scores of learning motivating factors based on the general fatigue (MFI-20) categories (Table 10).

A one-way ANOVA was conducted to compare the effect of fatigue on learning motivating factors. We analyzed learning motivating factors in four groups: minimal fatigue, mild fatigue, moderate fatigue, and severe fatigue. An analysis of variance revealed that the effect of general fatigue on individual attitude and expectation was significant, *F* (3, 402) = 2.628, *p* = 0.050. A Tukey post hoc test revealed that the scores of individual attitude and expectation were significantly higher in the moderate fatigue group (4.801, ±0.831) in comparison to the severe fatigue group (4.481, ±1.023) (*p* = 0.034).

An analysis of variance revealed that the effect of general fatigue on challenging goals was significant, *F* (3, 402) = 7.476, *p* < 0.001. A Tukey post hoc test revealed that the scores of challenging goals were significantly higher in the minimal fatigue group (4.778, ±1.112) in comparison to the moderate fatigue group (4.306, ±0.918) (*p* = 0.036) and the severe fatigue group (3.921, ±1.096) (*p* < 0.001). Additionally, the scores of challenging goals were significantly higher in the mild fatigue group (4.459, ±1.058) in comparison to the severe fatigue group (3.921, ±1.096) (*p* = 0.003).

An analysis of variance also revealed that the effect of general fatigue on social pressure and competition was significant, *F* (3, 402) = 6.162, *p* < 0.001. A Tukey post hoc test revealed that the scores of social pressure and competition were significantly higher in the mild fatigue group (3.687, ±1.255) in comparison to the severe fatigue group (3.053, ±1.270) (*p* = 0.001). Additionally, the scores of social pressure and competition were significantly higher in the moderate fatigue group (3.585, ±1.057) in comparison to the severe fatigue group (3.053, ±1.270) (*p* = 0.006).

However, there were no significant differences between the scores of clear direction *F* (3, 402) = 1.597, *p* = 0.189, reward and recognition *F* (3, 402) = 2.519, *p* = 0.058, or punishment, *F* (3, 402) = 1.363, *p* = 0.254, in different self-reported fatigue groups.

Next, to test hypothesis 4 (H4), presuming that extrinsic learning motivating factors predict increased anxiety, and intrinsic learning motivating factors predict decreased anxiety, we conducted a multiple linear regression using anxiety (GAD-7) as the criterion and learning motivating factors as predictors (Table 11) in groups of respondents, participating and not participating in e-learning-based computer programming courses.

A multiple linear regression model was calculated to predict anxiety based on learning motivating factors in groups of respondents participating and not participating in e-learning-based computer programming courses. A significant regression equation was found only in group of respondents participating in computer programming courses, *F* (6, 179) = 5.508, *p* < 0.001), with an *R*^2^ = 0.156. Respondents’ predicted anxiety was equal to 15.287 − 1.692 (challenging goals) points. Anxiety decreased −1.692 points for each challenging goals motivation point. Challenging goals (*B* = −1.692, *p* < 0.000) contributed significantly to the model and were significant predictors of diminished anxiety.

Furthermore, to test hypothesis 5 (H5), which assumed that extrinsic learning motivating factors predict increased depression, and intrinsic learning motivating factors predict decreased depression, we conducted a multiple linear regression using depression (PHQ-9) as the criterion and learning motivating factors as predictors (Table 12).

A multiple linear regression was also undertaken to predict depression based on learning motivating factors in groups of respondents participating and not participating in e-learning-based computer programming courses. A significant regression equation was found in group of respondents not participating in computer programming courses, *F* (6, 199) = 2.656, *p* = 0.017), with an *R*^2^ = 0.074. Respondents’ predicted depression was equal to 18.068 − 1.133 (challenging goals) points. Depression decreased −1.133 points for each challenging goals motivation point. Challenging goals (*B* = −1.133, *p* = 0.014) contributed significantly to the model and were significant predictors of diminished depression of respondents not participating in computer programming courses. Additionally, a significant regression equation was found in group of respondents participating in computer programming courses, *F* (6, 179) = 9.584, *p* < 0.001), with an *R*^2^ = 0.243. Computer programming learners’ predicted depression was equal to 24.130 − 2.233 (challenging goals) − 2.197 (clear direction) points. Depression decreased −2.233 points for each challenging goals motivation point and −2.197 points for each clear direction motivation point. Challenging goals (*B* = −2.233, *p* < 0.001) and clear direction (*B* = −2.197, *p* = 0.006) contributed significantly to the model and were significant predictors of diminished depression of participants of e-learning-based computer programming courses.

Additionally, to test hypothesis (H6), assuming that extrinsic learning motivating factors, anxiety, and depression predict increased general and mental fatigue, and intrinsic learning motivating factors predict decreased general and mental fatigue, we primarily conducted a multiple linear regression using general fatigue (MFI-20) as the criterion and learning motivating factors, anxiety, and depression as predictors (Table 13).

A multiple linear regression was also undertaken to predict general fatigue based on learning motivating factors, anxiety, and depression in groups of respondents participating and not participating in e-learning-based computer programming courses. A significant regression equation was found in the group of respondents not participating in computer programming courses, *F* (8, 197) = 7.058, *p* < 0.001), with an *R*^2^ = 0.223. Respondents’ predicted general fatigue was equal to 2.072 + 0.203 (individual attitudes and expectations) points + 0.061 (anxiety) + 0.033 (depression) points. Individual attitudes and expectations (*B* = 0.203, *p* = 0.049), anxiety (*B* = 0.061, *p* < 0.001), and depression (*B* = 0.033, *p* = 0.001) contributed significantly to the model and were significant predictors of general fatigue of respondents not participating in computer programming courses. Furthermore, a significant regression equation was found in the group of respondents participating in computer programming courses, *F* (8, 176) = 31.554, *p* < 0.001), with an *R*^2^ = 0.589. Computer programming learners’ predicted general fatigue was equal to 1.712 − 0.262 (individual attitudes and expectations) + 0.209 (clear direction) + 0.121 (reward and recognition) + 0.039 (anxiety) + 0.082 (depression) points. Individual attitudes and expectations (*B* = −0.262, *p* = 0.001), clear direction (*B* = 0.209, *p* = 0.019), reward and recognition (*B* = 0.121, *p* = 0.052), anxiety (*B* = 0.039, *p* = 0.004), and depression) (*B* = 0.082, *p* < 0.001) contributed significantly to the model and were significant predictors of general fatigue of participants of e-learning-based computer programming courses.

Then, we conducted a multiple linear regression using mental fatigue as the criterion and learning motivating factors, anxiety, and depression as predictors (Table 14).

A multiple linear regression was also undertaken to predict mental fatigue based on learning motivating factors, anxiety, and depression in groups of respondents participating and not participating in e-learning-based computer programming courses. A significant regression equation was found in the group of respondents not participating in computer programming courses, *F* (8, 197) = 5.229, *p* < 0.001), with an *R*^2^ = 0.175. Respondents’ predicted mental fatigue was equal to 3.247 + 0.041 (anxiety) + 0.026 (depression) points. Anxiety (*B* = 0.041, *p* < 0.001) and depression (*B* = 0.026, *p* = 0.011) contributed significantly to the model and were significant predictors of mental fatigue of respondents not participating in computer programming courses. Furthermore, a significant regression equation was found in the group of respondents participating in computer programming courses, *F* (8, 176) = 25.338, *p* < 0.001), with an *R*^2^ = 0.535. Computer programming learners’ predicted mental fatigue was equal to 2.451 − 0.252 (challenging goals) − 0.095 (punishment) + 0.092 (social pressure and competition) + 0.073 (depression) points. Challenging goals (*B* = −0.252, *p* < 0.001), punishment (*B* = −0.095, *p* = 0.028), social pressure and competition (*B* = 0.092, *p* = 0.055), and depression (*B* = 0.073, *p* < 0.001) contributed significantly to the model and were significant predictors of mental fatigue of participants of e-learning-based computer programming courses.

**Figure 1:**
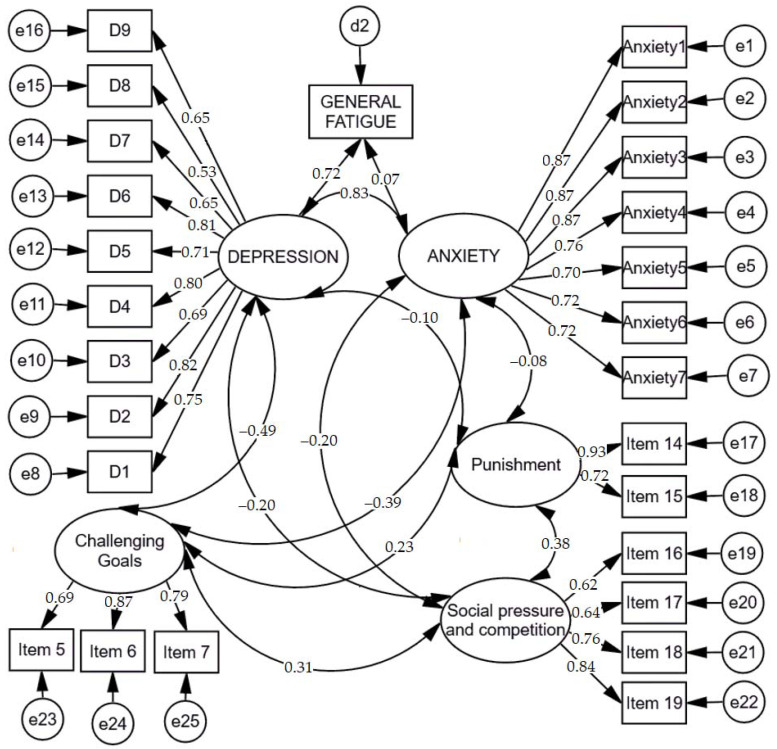
Standardized results on the model of associations between learning motivating factors (challenging goals, punishment, social pressure, and competition), anxiety, depression, and fatigue in the group of participants of e-learning-based computer programming courses.

Based on the literature review and previous analyses that indicate the differences and impact of several learning motivation factors, to test hypothesis 7 (H7), assuming that associations exist between learning motivating factors, anxiety, depression, and fatigue, but they differ between participants and non-participants of e-learning-based computer programming courses, we first created a model of associations between the learning motivating factors (challenging goals, punishment, social pressure, and competition), anxiety, depression, and fatigue in the group of participants of e-learning-based computer programming courses. Standardized results of the model are presented in Figure 1. To assess the model fit, the Comparative Fit Index (CFI), the Tucker–Lewis coefficient (TLI), and Root Mean Square Error of Approximation (RMSEA) were used. Findings revealed that the fit of the model was acceptable, χ^2^ = 558.241; *df* = 288; *p* < 0.001; CFI = 0.904; TLI = 0.906; RMSEA = 0.071 [0.062–0.079]. 

Scalar estimates of the model of associations between learning motivating factors, anxiety, depression, and fatigue in the sample of participants of e-learning-based computer programming courses are displayed in Table 15.

**Figure 2:**
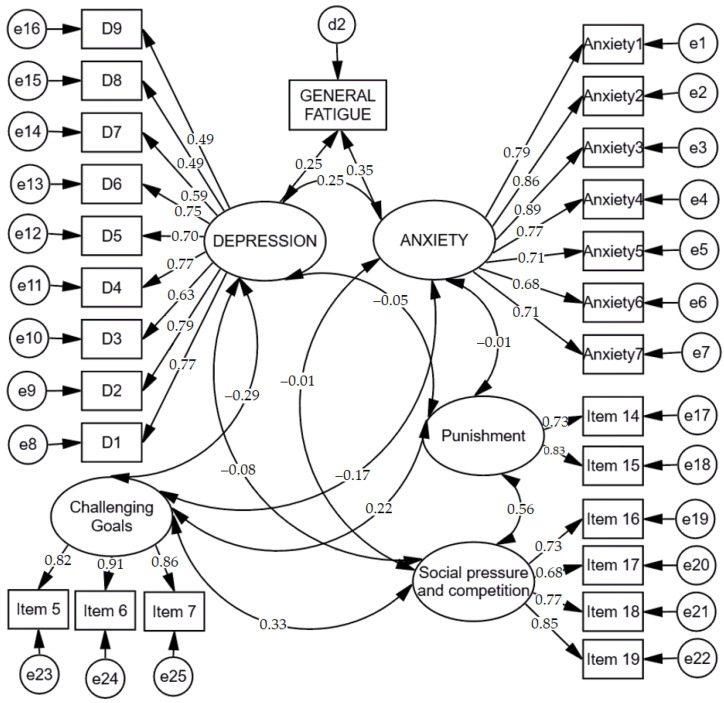
Standardized results of the model of associations between learning motivating factors (challenging goals, punishment, social pressure, and competition), anxiety, depression, and fatigue in the group of respondents who did not participate in e-learning-based computer programming courses.

Furthermore, we created a model of associations between some learning motivating factors (challenging goals, punishment, social pressure, and competition), anxiety, depression, and fatigue in the group of those who did not participate in e-learning-based computer programming courses. Standardized results of the model are presented in Figure 2. To assess the model fit, the Comparative Fit Index (CFI), the Tucker–Lewis coefficient (TLI), and Root Mean Square Error of Approximation (RMSEA) were used. Findings revealed that the fit of the model was acceptable, χ^2^ = 563.004; *df* = 288; *p* < 0.001; CFI = 0.908; TLI = 0.910; RMSEA = 0.061 [0.054–0.069].

Scalar estimates of the model of associations between learning motivating factors, anxiety, depression, and fatigue in the sample of non-participants of e-learning-based computer programming courses are displayed in Table 16.

**Figure 3:**
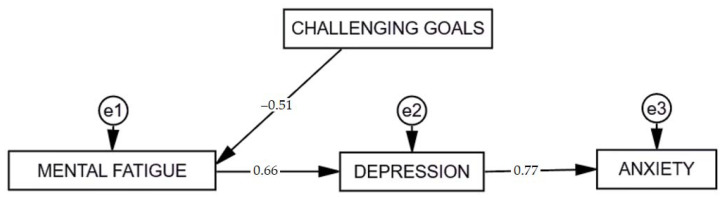
Path diagram: challenging goals, mental fatigue, depression, and anxiety in participants of e-learning-based computer programming courses.

Furthermore, based on the results of multiple regression analysis, structural equation modeling (SEM) was conducted to analyze the associations between challenging goals, mental fatigue, depression, and anxiety in the group of participants of e-learning-based computer programming courses. The path diagram is displayed in Figure 3.

Scalar estimates of the model are presented in Table 17. Findings revealed that the fit of the model was acceptable, χ^2^ (3) = 8.147; *p* = 0.043; CFI = 0.984; TLI = 0.969; NFI = 0.976; RMSEA = 0.096 [0.015–0.179]; SRMR = 0.052.

**Figure 4:**
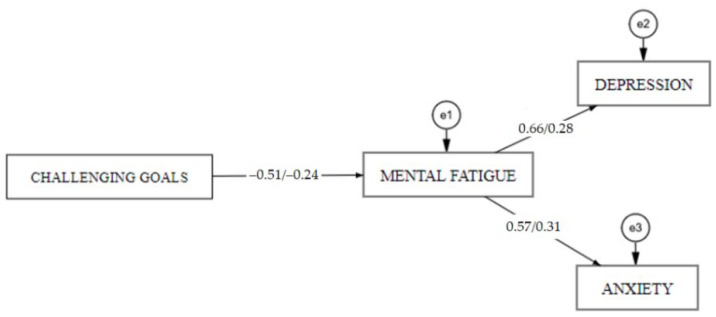
Alternative path diagrams (challenging goals, mental fatigue, depression, and anxiety) in groups of participants and non-participants in e-learning-based computer programming courses.

To examine alternative models of associations between challenging goals, mental fatigue, depression, anxiety, and assess differences between participants and non-participants of e-learning-based computer programming courses, we conducted structural equation modelling with two sample groups. Respondents of group 1 were not participating in e-learning-based program, and respondents of group 2 were participating in computer programming courses. The diagram of the path is displayed in Figure 4.

The results of SEM analysis are presented in Table 18. SEM analysis showed that the motivational factor challenging goals statistically significantly predicts mental fatigue, and mental fatigue statistically significantly predicts depression and anxiety in both sample groups. In Group 2, challenging goals explains 25.7% of the variance of mental fatigue, and mental fatigue explains 44.1% of the variance of depression and 31.1% of the variance of anxiety. In group 1, challenging goals explains 5.5% of the variance of mental fatigue, and mental fatigue explains 7.7% of the variance of depression and 9.6% of the variance of anxiety. Findings revealed that the fit of the model was acceptable, χ^2^ (4) = 14.858; *p* = 0.005; CFI = 0.972; TLI = 0.916; NFI = 0.963; RMSEA = 0.118 [0.058–0.185]; SRMR = 0.054.

## 4. Discussion

This study was the first to explore associations between depression, anxiety, fatigue, and learning motivating factors in e-learning-based education, including computer programming learning, during the COVID-19 pandemic. The examination of learning motivation was based on a model of extrinsic and intrinsic learning motivating factors, developed by Law et al. [6]. This research was conducted during the COVID-19 pandemic. The context of the COVID-19 quarantine can be characterized by the rapid implementation of e-learning [93], which helped us compare the motivating factors in e-learning-based computer programming education and e-learning in education other than computer programming. The pandemic context can also be characterized by the statistically significant increase in the rates of anxiety and depression [65,70,72,73,74], chronic fatigue [66], and psychological distress [67]. Due to these conditions, the research questions were extremely important, because previous studies indicated that the states of severe anxiety and depression relate to dramatically diminished e-learning motivation, or even its absence [76]. 

### 4.1. Lower Scores of Depression Partially Relate to Higher Scores of Learning Motivating Factors 

In this study, we assumed that lower scores of depression relate to higher scores of learning motivating factors. Thus, we compared the scores of learning motivating factors based on the PHQ-9 categories. The results partially confirmed this hypothesis and showed that the scores of the motivating factor individual attitude and expectation were significantly higher in the minimal depression group in comparison to the moderately severe depression group. The scores of challenging goals were significantly higher in the minimal depression group in comparison to the mild depression, moderate depression, moderately severe depression, and severe depression groups, and the scores of challenging goals were significantly higher in the mild depression group in comparison to the moderately severe depression group. The scores of clear direction were significantly higher in the minimal depression group in comparison to the moderately severe depression or severe depression groups, and the scores of clear direction were significantly higher in the mild depression group in comparison to the moderately severe depression group and the severe depression group. The scores of social pressure and competition were significantly higher in the mild depression group in comparison to the moderately severe depression group. However, there were no significant differences in the scores of reward and recognition and the scores of punishment in the depression groups. To summarize, the scores of individual attitude and expectation, challenging goals, clear direction, social pressure, and competition varied between the depression categories. However, no significant differences were identified in the scores of reward and recognition and punishment in the different depression-level groups. These results align with previous research suggesting that depression is associated with learning difficulties, both behavioral and neural, partly due to impaired generation and updating of outcome predictions [94]. Moreover, these findings support studies implying that learning motivation can be impacted by depression [95,96,97,98].

### 4.2. Lower Anxiety Scores Partially Relate to Higher Scores of Intrinsic Learning Motivating Factors

Furthermore, we presumed that lower anxiety scores relate to higher intrinsic, but not extrinsic, motivating factors. Hence, we compared the scores of learning motivating factors based on the GAD-7 categories. The results partially confirmed this hypothesis and showed that the scores of challenging goals were significantly higher in the minimal anxiety group than the moderate anxiety and severe anxiety groups. Challenging goals were significantly higher in the mild anxiety group in comparison to the severe anxiety group; the scores of social pressure and competition were significantly higher in the minimal anxiety group in comparison to the severe anxiety group, and the scores of social pressure and competition were significantly higher in the mild anxiety group in comparison to the severe anxiety group. However, there were no significant differences in individual attitude and expectation, reward and recognition, and punishment scores between different anxiety groups. To summarize, in this study, the scores of challenging goals, and social pressure and competition, significantly varied between different anxiety categories. However, no significant differences were identified in the scores of individual attitude and expectation, reward and recognition, and punishment between different anxiety-level groups. These findings partially contradict some previous results evidencing that anxiety is unrelated to learning outcomes and motivation in computer programming learning [37,38,39]. However, the findings support those of other studies, which demonstrated the impact of anxiety on learning motivation [76,99,100], and the impact of learning motivation on emotions [25,26,27].

### 4.3. Lower Scores of General Fatigue Partially Relate to Higher Scores of Learning Motivating Factors 

Next, we assumed that lower scores of general fatigue relate to higher scores of learning motivating factors. Therefore, we compared the scores of learning motivating factors based on the general fatigue (MFI-20) categories. The results partially confirmed this hypothesis and showed that the scores of individual attitude and expectation were significantly higher in the moderate fatigue group in comparison to the severe fatigue group. The scores of challenging goals were significantly higher in the minimal fatigue group in comparison to the moderate fatigue group and the severe fatigue group, and the scores of challenging goals were significantly higher in the mild fatigue group in comparison to the severe fatigue group. The scores of social pressure and competition were significantly higher in the mild fatigue group in comparison to the severe fatigue group, and the scores of social pressure and competition were significantly higher in the moderate fatigue group in comparison to the severe fatigue group. However, there were no significant differences in the scores of clear direction, reward and recognition, and punishment between different self-reported fatigue-level groups. To summarize, this study demonstrated that the scores of individual attitude and expectation, challenging goals, social pressure, and competition varied across different fatigue categories, but no significant differences were identified in the scores of clear direction, reward and recognition, and punishment between different fatigue level-groups. These results partially confirm the findings of other authors, evidencing associations between fatigue and learning motivation [101,102,103,104,105,106]. However, it is unclear whether learners’ fatigue was related to learning burnout or the psychologically challenging COVID-19 pandemic, and why some motivational factors, for example, striving for reward and recognition or fear of punishment, did not contribute to the increased fatigue.

### 4.4. Learning Motivating Factors Partially Predict Anxiety Level

Next, we presumed that extrinsic learning motivating factors predict increased anxiety, and intrinsic learning motivating factors predict decreased anxiety. Thus, we conducted a multiple linear regression using anxiety (GAD-7) as the criterion and learning motivating factors as predictors in groups of respondents participating and not participating in e-learning-based computer programming courses. The results partially confirmed this hypothesis and showed that in the group of respondents participating in computer programming courses, challenging goals was a significant predictor of diminished anxiety. To summarize, this study showed that intrinsic the motivating factor challenging goals predicts decreased anxiety in a group of participants of e-learning-based computer programming courses. These findings are, to some extent, in line with those of previous research, suggesting that intrinsic learning motivation is related to better learning outcomes and higher satisfaction with the learning process, whereas extrinsic learning motivation is related to higher stress levels [35,36,67]. However, it is partially clear why only one intrinsic motivational factor (challenging goals) contributed to the decreased anxiety [107], and why this pattern was not apparent in the group of other e-learners, which would be in line with many other studies [33,107,108,109,110,111]. 

### 4.5. Learning Motivating Factors Partially Predict Depression Level

Furthermore, we assumed that extrinsic learning motivating factors predict increased depression, and intrinsic learning motivating factors predict decreased depression. Thus, we conducted a multiple linear regression using depression (PHQ-9) as the criterion and learning motivating factors as predictors in groups of respondents participating and not participating in e-learning-based computer programming courses. To a small extent, The results confirmed this hypothesis and showed that, in the group of respondents who did not participate in computer programming courses, the challenging goals factor was a significant predictor of diminished depression. However, the motivating intrinsic factor of challenging goals and the extrinsic factor of clear direction were significant predictors of diminished depression in the group of participants of e-learning-based computer programming courses. To summarize, this research demonstrated that the extrinsic motivating factor of clear direction and the intrinsic motivating factor of challenging goals predict decreased depression in the group of participants of e-learning-based computer programming courses, whereas the intrinsic motivating factor of challenging goals predicts decreased depression in the group of other e-learners. These findings are moderately supported by previous research suggesting the benefits of some intrinsic and extrinsic motivating factors [32,33,107,111,112,113]. However, the added value of clear direction and challenging goals for computer programming learners needs further investigation.

### 4.6. Learning Motivating Factors, Anxiety, and Depression Partially Predict Fatigue

Additionally, we assumed that extrinsic learning motivating factors, anxiety, and depression predict increased general and mental fatigue, and intrinsic learning motivating factors predict decreased general and mental fatigue. Thus, we conducted a multiple linear regression using general and mental fatigue (MFI-20) as the criteria and learning motivating factors, anxiety, and depression as predictors. The results, to some extent, confirmed this hypothesis and showed that individual attitudes and expectations, anxiety, and depression predicted higher general fatigue of respondents who did not participate in e-learning-based computer programming courses. Interestingly, the computer programming learners’ intrinsic motivating factor of individual attitude and expectation predicted diminished general fatigue; however, clear direction, reward and recognition, anxiety, and depression were significant predictors of increased general fatigue of participants of e-learning-based computer programming courses. Furthermore, anxiety and depression were significant predictors of mental fatigue of respondents not participating in computer programming courses. Remarkably, the computer programming learners’ intrinsic motivating factor of challenging goals and their extrinsic motivating factor of punishment predicted diminished mental fatigue; however, social pressure and competition, and depression, were significant predictors of increased mental fatigue of participants of e-learning-based computer programming courses. To summarize, this study found that the intrinsic motivating factor of individual attitude and expectation predicted diminished general fatigue, and clear direction, reward and recognition, anxiety, and depression predicted increased general fatigue of participants of e-learning-based computer programming courses. Individual attitudes and expectations, anxiety, and depression predicted higher general fatigue of non-participants; the computer programming learners’ intrinsic motivating factor of challenging goals and their extrinsic motivating factor of punishment predicted diminished mental fatigue, whereas the mental fatigue of other e-learners was predicted only by anxiety and depression. Therefore, this research modestly contributed to a better understanding of the complexity of associations between intrinsic and extrinsic learning motivating factors, anxiety, depression, and fatigue, as analyzed by other researchers [114,115,116,117,118,119]. However, it is unclear why computer programming e-learners’ individual attitude and expectation predicted diminished general fatigue, whereas other e-learners’ individual attitude and expectation predicted an increase in general fatigue. This question needs further investigation. 

### 4.7. Associations between the Study Variables Partially Differ in the Compared Groups 

Based on a literature review and previous analyses, which suggested the differences and impact of several learning motivation factors, we assumed that associations exist between learning motivating factors, anxiety, depression, and fatigue, and that these associations differ between participants and non-participants of e-learning-based computer programming courses. Thus, we tested several models of associations between the study variables. This study partially confirmed the hypothesis and identified several possible paths and models of associations between learning motivating factors (challenging goals, punishment, social pressure, and competition), anxiety, depression, and fatigue in groups of participants and non-participants of e-learning-based computer programming courses. However, although some differences in associations between the groups were identified, the SEM analyses demonstrated that the intrinsic motivating factor of challenging goals statistically significantly predicts lower mental fatigue, and mental fatigue statistically significantly predicts depression and anxiety in both groups of participants and non-participants of e-learning-based computer programming courses. To summarize, this study demonstrated that associations between learning motivating factors, anxiety, depression, and fatigue differ between participants and non-participants of e-learning-based computer programming courses. However, the intrinsic motivating factor of challenging goals statistically significantly predicts lower mental fatigue, whereas mental fatigue statistically significantly predicts depression and anxiety in both sample groups. These findings are consistent with many previous studies that suggest the added value of intrinsic learning motivation [33,99,107,108,109,110,111]. In the future, it will be essential that the identification of the associations between learning motivating factors and the positive states receives increased attention from researchers [120,121].

### 4.8. Limitations and Future Directions

Several limitations to this study can be mentioned. First, because the mental health issues in e-learning-based education are under-researched, this study lacks a solid theoretical basis. Second, because the objective health status was not measured, bias may have occurred due to the use of self-reported measures only, and the omission of objective indicators. Third, the findings should be regarded with caution, considering that the data were collected online. Fourth, the research samples were not representative but random, suggesting the necessity to analyze representative samples of e-learners; thus, generalizations should be made with caution. Fifth, although the sample size satisfied the minimal requirements for the applied statistical models, and the data fit was acceptable, the results should be regarded cautiously due to the relatively small sample size. Sixth, some possible theoretical bias may have occurred in the selected model. Furthermore, this study was conducted in Lithuania, and the results may reflect the specifics of this area, suggesting the necessity to analyze the impact of cultural factors, considering the more specific aspects of each culture. Finally, the findings suggest a necessity for longitudinal or experimental research design, because, based on the data obtained, it is possible to only identify the existence of significant relationships among the examined variables. Thus, the conclusions should be regarded with caution, especially regarding causality. Although this study found that higher scores of individual attitude and expectation, challenging goals, clear direction, social pressure, and competition are associated with better mental health, the causality issues need further investigation, because reverse causality is also likely to occur.

### 4.9. Theoretical Implications

From a theoretical perspective, this study was the first of its kind to explore the associations between depression, anxiety, fatigue, and learning motivating factors in e-learning-based education, including computer programming learning, during the COVID-19 pandemic. Many learning motivation models exist, but this study was based on a theory of extrinsic and intrinsic learning motivating factors, developed by Law et al. [6]. Although the findings revealed the complexity of the relations between learning motivating factors, anxiety, depression, and fatigue, numerous questions remained unanswered. It is unclear whether learners’ fatigue relates to learning burnout, or was a consequence of the psychologically challenging COVID-19 pandemic. In addition, it is unclear why striving for reward and recognition or fear of punishment did not increase fatigue. Furthermore, it is only partially clear why only one intrinsic motivational factor (challenging goals) contributed to the decreased anxiety and why this pattern was not apparent in the group other than computer programming e-learners. The emotional benefits of clear direction and challenging goals for computer programming learners also need further investigation. It is also unclear why computer programming e-learners’ individual attitude and expectation predicted diminished general fatigue, whereas other e-learners’ individual attitude and expectation predicted an increase in general fatigue. In the future, it would be interesting to identify the associations between intrinsic and extrinsic learning motivating factors and the positive states such as flow or zest, which have recently received the increased attention of researchers [120,121].

### 4.10. Practical Implications

The COVID-19 quarantine forced the rapid implementation of e-learning [93]. The psychologically challenging pandemic also increased the rates of anxiety, depression [65,70,72,73,74], and fatigue [66]. Because severe anxiety and depression states relate to dramatically diminished e-learning motivation, or even its absence [76], it was important to identify e-learning motivating factors related to mental health under the challenging psychological circumstances of the COVID-19 pandemic. Because computer programming skills are a core competency that professionals are expected to possess in the era of rapid technology development [1], it was also essential to identify the specific factors related to computer programming learning. This research revealed that mental health, namely, depression, anxiety, and fatigue, are related to the scores of intrinsic and extrinsic learning motivating factors. In addition, the motivating factor of challenging goals was strongly related to better mental health in groups of participants and non-participants of e-learning-based computer programming courses. This implies that e-learners’ good mental health is a target for both health and education policy makers, health professionals, and educators. 

## 5. Conclusions

This study aimed to identify associations between depression, anxiety, fatigue, and learning motivating factors in participants and non-participants of e-learning-based computer programming courses. It was found that the scores of individual attitude and expectation, challenging goals, clear direction, social pressure, and competition varied between the depression categories. However, no significant differences were identified in the scores of reward and recognition, and punishment, in the different depression-level groups. The scores of challenging goals, and social pressure and competition, significantly varied between the anxiety categories, but no significant differences were identified in the scores of individual attitude and expectation, reward and recognition, and punishment between different anxiety-level groups. The scores of individual attitude and expectation, challenging goals, and social pressure and competition varied across different fatigue categories, but no significant differences were identified in the scores of clear direction, reward and recognition, and punishment between different fatigue-level groups. The intrinsic motivating factor of challenging goals predicted decreased anxiety in a group of participants of e-learning-based computer programming courses. The extrinsic motivating factor of clear direction and the intrinsic motivating factor of challenging goals predicted decreased depression in a group of participants of e-learning-based computer programming courses, and the intrinsic motivating factor of challenging goals predicted decreased depression in a group of other e-learners. This study found that the intrinsic motivating factor of individual attitude and expectation predicted diminished general fatigue, and clear direction, reward and recognition, anxiety, and depression predicted increased general fatigue of participants of e-learning-based computer programming courses. Individual attitude and expectation, anxiety, and depression predicted higher general fatigue of non-participants. The computer programming learners’ intrinsic motivating factor of challenging goals and their extrinsic motivating factor of punishment predicted diminished mental fatigue, whereas the mental fatigue of other e-learners was predicted only by anxiety and depression. Associations between learning motivating factors, anxiety, depression, and fatigue differed between participants and non-participants of e-learning-based computer programming courses. However, the intrinsic motivating factor of challenging goals statistically significantly predicted lower mental fatigue, whereas mental fatigue statistically significantly predicted depression and anxiety in both sample groups. Nevertheless, the results of this particular study should be regarded with caution due to the relatively small sample size, the possible biases in the selected theoretical and statistical models, the research design, and other limitations.

## Figures and Tables

**Table 1:** Cronbach alphas for the Patient Health Questionnaire-9 (PHQ-9), the Generalized Anxiety Disorder Scale-7 (GAD-7), the Multidimensional Fatigue Inventory-20 (MFI-20), and the Learning Motivating Factors Questionnaire.

Scales and Subscales	Cronbach Alpha
GAD-7	0.915
PHQ-9	0.889
MFI-20	0.933
General Fatigue	0.823
Physical Fatigue	0.869
Reduced Activity	0.784
Reduced Motivation	0.671
Mental Fatigue	0.854
Learning Motivating Factors Questionnaire (LMF)	0.868
Individual Attitude and Expectation	0.811
Challenging Goals	0.866
Clear Direction	0.698
Reward and Recognition	0.743
Punishment	0.778
Social pressure and competition	0.824

**Table 2:** The frequencies of the PHQ-9 self-reported categories.

PHQ-9	Frequency	Percent	95% CI	*p*
Minimal depression (0–4)	108	24.3	[2.06–2.61]	<0.001
Mild depression (5–9)	130	29.3	[6.71–7.20]	<0.001
Moderate depression (10–14)	74	16.7	[11.62–12.33]	<0.001
Moderately severe depression (15–19)	50	11.3	[16.31–17.05]	<0.001
Severe depression (20–27)	32	7.2	[21.33–22.86]	<0.001

CI, confidence interval.

**Table 3:** The frequencies of the GAD-7 self-reported categories.

GAD-7	Frequency	Percent	95% CI	*p*
Minimal anxiety (0–4)	161	36.3	[1.98–2.42]	<0.001
Mild anxiety (5–9)	151	34	[6.41–6.85]	<0.001
Moderate anxiety (10–14)	73	16.4	[11.43–12.11]	<0.001
Severe anxiety (15–21)	53	11.9	[17.36–18.46]	<0.001

**Table 4:** The frequencies of the MFI-20 self-reported categories.

MFI-20: General Fatigue	Frequency	Percent	95% CI	*p*
Minimal fatigue (4–7)	53	11.9	[5.56–6.21]	<0.001
Mild fatigue (8–11)	133	30	[9.49–9.87]	<0.001
Moderate fatigue (12–15)	168	37.8	[13.31–13.65]	<0.001
Severe fatigue (16–20)	90	20.3	[16.99–17.59]	<0.001

**Table 5:** The MFI-20: descriptive statistics and correlations between the subscales.

MFI-20 Variables	*M*	*SD*	1	2	3	4
Physical fatigue	2.813	1.030	-			
Reduced activity	2.988	0.905	0.630 ***	-		
Reduced motivation	2.512	0.790	0.569 ***	0.729 ***	-	
Mental fatigue	2.846	0.911	0.416 ***	0.647 ***	0.631 ***	-
General fatigue	3.052	0.934	0.748 ***	0.661 ***	0.615 ***	0.545 ***

*M*, mean; *SD*, standard deviation. *** *p* < 0.001.

**Table 6:** The Learning Motivating Factors Questionnaire: descriptive statistics and correlations between the subscales.

Learning Motivation Variables	*M*	*SD*	1	2	3	4	5
Individual attitude and expectation	4.71	0.86	-				
Challenging goals	4.33	1.08	0.407 ***	-			
Clear direction	4.97	0.78	0.648 ***	0.449 ***	-		
Reward and recognition	4.85	0.90	0.546 ***	0.118 *	0.504 ***	-	
Punishment	3.46	1.33	0.230 ***	0.158 **	0.257 ***	0.161 **	-
Social pressure and competition	3.46	1.21	0.295 ***	0.284 ***	0.199 ***	0.230 ***	0.422 ***

* *p* < 0.05; ** *p* < 0.01; *** *p* < 0.001.

**Table 7:** The PHQ-9 and the GAD-7: descriptive statistics and correlations between the scales.

Scales	*M*	*SD*	1
PHQ-9	9.094	6.240	-
GAD-7	7.221	5.378	0.499 ***

*** *p* < 0.001.

**Table 8:** Differences in learning motivating factors based on the PHQ-9 categories.

Learning Motivating Factors	Depression Categories	*N*	Mean	Standard Deviation	Standard Error	95% Confidence Interval for Mean	
						Lower *B*	Upper *B*
Individual attitude and expectation	Minimal depression	108	4.859	0.783	0.075	4.709	5.008
	Mild depression	130	4.764	0.818	0.071	4.621	4.905
	Moderate depression	73	4.678	0.848	0.099	4.480	4.876
	Moderately severe depression	49	4.403	0.904	0.129	4.143	4.662
	Severe depression	32	4.445	1.114	0.197	4.043	4.846
Challenging goals	Minimal depression	108	4.833	0.808	0.077	4.679	4.988
	Mild depression	130	4.354	1.091	0.095	4.164	4.543
	Moderate depression	73	4.073	1.026	0.120	3.833	4.312
	Moderately severe depression	49	3.708	1.079	0.154	3.398	4.018
	Severe depression	32	3.844	1.173	0.207	3.420	4.267
Clear direction	Minimal depression	108	5.172	0.737	0.070	5.032	5.313
	Mild depression	130	5.015	0.719	0.063	4.890	5.140
	Moderate depression	73	4.995	0.748	0.087	4.820	5.170
	Moderately severe depression	49	4.612	0.681	0.097	4.417	4.808
	Severe depression	32	4.583	1.057	0.187	4.202	4.965
Reward and recognition	Minimal depression	108	4.811	0.971	0.093	4.626	4.997
	Mild depression	130	4.917	0.809	0.071	4.778	5.058
	Moderate depression	73	4.954	0.863	0.101	4.753	5.156
	Moderately severe depression	49	4.776	0.989	0.141	4.491	5.060
	Severe depression	32	4.646	0.939	0.166	4.307	4.984
Punishment	Minimal depression	108	3.435	1.354	0.130	3.177	3.693
	Mild depression	130	3.527	1.203	0.106	3.318	3.736
	Moderate depression	73	3.596	1.384	0.162	3.273	3.919
	Moderately severe depression	49	2.980	1.342	0.192	2.594	3.365
	Severe depression	32	3.516	1.511	0.267	2.971	4.060
Social pressure and competition	Minimal depression	108	3.495	1.170	0.113	3.272	3.719
	Mild depression	130	3.604	1.176	0.103	3.400	3.808
	Moderate depression	73	3.500	1.187	0.139	3.223	3.777
	Moderately severe depression	49	2.975	1.198	0.171	2.630	3.319
	Severe depression	32	3.297	1.418	0.251	2.786	3.808

**Table 9:** Differences in learning motivating factors based on the GAD-7 categories.

Learning Motivating Factors	Anxiety Categories	*N*	Mean	Standard Deviation	Standard Error	95% Confidence Interval for Mean	
						Lower *B*	Upper *B*
Individual attitude and expectation	Minimal anxiety	148	4.797	0.832	0.068	4.662	4.932
	Mild anxiety	137	4.725	0.885	0.076	4.575	4.874
	Moderate anxiety	71	4.585	0.828	0.982	4.389	4.781
	Severe anxiety	49	4.612	0.920	0.131	4.348	4.877
Challenging goals	Minimal anxiety	148	4.604	1.020	0.084	4.438	4.516
	Mild anxiety	137	4.329	1.112	0.095	4.140	4.516
	Moderate anxiety	71	4.155	0.946	0.112	3.931	4.378
	Severe anxiety	49	3.782	1.154	0.165	3.450	4.114
Clear direction	Minimal anxiety	148	5.045	0.763	0.063	4.921	5.169
	Mild anxiety	137	5.059	0.759	0.065	4.930	5.187
	Moderate anxiety	71	4.826	0.756	0.090	4.647	5.005
	Severe anxiety	49	4.728	0.889	0.127	4.472	4.983
Reward and recognition	Minimal anxiety	148	4.746	0.925	0.076	4.595	4.896
	Mild anxiety	137	4.966	0.883	0.075	4.817	5.115
	Moderate anxiety	71	4.859	0.861	0.102	4.655	5.063
	Severe anxiety	49	4.803	0.902	0.133	4.536	4.935
Punishment	Minimal anxiety	148	3.449	1.326	0.109	3.234	3.665
	Mild anxiety	137	3.533	1.320	0.113	3.310	3.756
	Moderate anxiety	71	3.521	1.291	0.153	3.216	3.827
	Severe anxiety	49	3.214	1.331	0.066	3.332	3.592
Social pressure and competition	Minimal anxiety	148	3.525	1.127	0.093	3.342	3.708
	Mild anxiety	137	3.564	1.327	0.113	3.340	3.788
	Moderate anxiety	71	3.450	1.090	0.129	3.192	3.709
	Severe anxiety	49	3.010	1.187	0.170	2.670	3.581

**Table 10:** Differences in learning motivating factors based on the MFI-20 categories.

Learning Motivating Factors	Fatigue Categories	*N*	Mean	Standard Deviation	Standard Error	95% Confidence Interval for Mean	
						Lower *B*	Upper *B*
Individual attitude and expectation	Minimal fatigue	48	4.781	0.844	0.122	4.536	5.026
	Mild fatigue	122	4.730	0.848	0.077	4.578	4.881
	Moderate fatigue	156	4.801	0.831	0.067	4.670	4.933
	Severe fatigue	80	4.481	0.918	0.103	4.277	4.686
Challenging goals	Minimal fatigue	48	4.778	1.112	0.160	4.455	5.100
	Mild fatigue	122	4.459	1.058	0.096	4.269	4.649
	Moderate fatigue	156	4.306	1.023	0.082	4.143	4.467
	Severe fatigue	80	3.921	1.096	0.123	3.677	4.416
Clear direction	Minimal fatigue	48	5.076	0.951	0.137	4.800	5.352
	Mild fatigue	122	5.071	0.699	0.063	4.946	5.196
	Moderate fatigue	156	4.915	0.804	0.644	4.787	5.042
	Severe fatigue	80	4.879	0.741	0.829	4.714	5.044
Reward and recognition	Minimal fatigue	48	4.542	1.104	0.159	4.221	4.862
	Mild fatigue	122	4.962	0.821	0.744	4.815	5.109
	Moderate fatigue	156	4.853	0.899	0.072	4.7104	4.995
	Severe fatigue	80	4.858	0.873	0.098	4.664	5.053
Punishment	Minimal fatigue	48	3.229	1.313	0.189	2.848	3.610
	Mild fatigue	122	3.610	1.373	0.124	3.365	3.857
	Moderate fatigue	156	3.490	1.249	0.100	3.293	3.688
	Severe fatigue	80	3.313	1.415	0.158	2.998	3.627
Social pressure and competition	Minimal fatigue	48	3.172	1.244	0.180	2.811	3.533
	Mild fatigue	122	3.687	1.255	0.114	3.418	3.911
	Moderate fatigue	156	3.585	1.057	0.085	3.418	3.752
	Severe fatigue	80	3.053	1.270	0.142	2.770	3.336

**Table 11:** Multiple regression models in which the dependent variable is anxiety and the predictors are learning motivating factors.

Model	Non-Standardized Coefficients		Standardized Coefficients	*t*	Significance
	*B*	Standard Error	Beta		
A. Respondents do not participate in e-learning-based computer programming courses					
(Constant)	12.244	2.655		4.612	0.000
Individual attitude and expectation	−0.065	0.642	−0.010	−0.102	0.919
Challenging goals	−0.592	0.401	−0.126	−1.477	0.141
Clear direction	−0.581	0.661	−0.085	−0.879	0.381
Reward and recognition	0.033	0.543	0.005	0.060	0.952
Punishment	0.037	0.301	0.010	0.124	0.901
Social pressure and competition	0.141	0.347	0.033	0.405	0.686
*R* = 0.181; *R* Square = 0.033; Adjusted *R* Square = 0.005; Standard Error of the Estimate = 5.24862; *F* (6, 212) = 1.192, *p* = 0.312					
B. Respondents participate in e-learning-based computer programming courses					
(Constant)	15.287	2.747		5.565	0.000
Individual attitude and expectation	0.245	0.608	0.040	0.404	0.687
Challenging goals	−1.692	0.417	−0.318	−4.061	0.000
Clear direction	−0.935	0.702	−0.134	−1.331	0.185
Reward and recognition	0.783	0.499	0.131	1.570	0.118
Punishment	0.185	0.330	0.043	0.561	0.576
Social pressure and competition	−0.431	0.364	−0.091	−1.183	0.239
*R* = 0.395; *R* Square = 0.156; Adjusted *R* Square = 0.128; Standard Error of the Estimate = 5.16559; *F* (6, 179) = 5.508, *p* < 0.001					

**Table 12:** Multiple regression models in which the dependent variable is depression and the predictors are learning motivating factors.

Model	Non-Standardized Coefficients		Standardized Coefficients	*t*	Significance
	*B*	Standard Error	Beta		
A. Respondents do not participate in e-learning-based computer programming courses					
(Constant)	18.068	3.077		5.872	0.000
Individual attitude and expectation	−0.192	0.747	−0.026	−0.257	0.797
Challenging goals	−1.133	0.459	−0.211	−2.469	0.014
Clear direction	−0.464	0.758	−0.059	−0.612	0.541
Reward and recognition	−0.271	0.641	−0.038	−0.423	0.673
Punishment	0.175	0.346	0.040	0.507	0.613
Social pressure and competition	−0.014	0.394	-0.003	−0.036	0.972
*R* = 0.272; *R* Square = 0.074; Adjusted *R* Square = 0.046; Stadard Error of the Estimate = 5.86205; *F* (6, 199) = 2.656, *p* = 0.017					
B. Respondents participate in e-learning-based computer programming courses					
(Constant)	24.130	3.065		7.873	0.000
Individual attitude and expectation	0.370	0.677	0.052	0.546	0.586
Challenging goals	−2.233	0.464	−0.357	−4.809	0.000
Clear direction	−2.197	0.783	−0.268	−2.806	0.006
Reward and recognition	0.874	0.554	0.125	1.578	0.116
Punishment	0.335	0.368	0.066	0.911	0.363
Social pressure and competition	−0.478	0.407	−0.085	−1.176	0.241
*R* = 0.493; *R* Square = 0.243; Adjusted *R* Square = 0.218; Standard Error of the Estimate = 5.75620; *F* (6, 179) = 9.584, *p* < 0.001					

**Table 13:** Multiple regression models in which the dependent variable is general fatigue and the predictors are learning motivating factors, anxiety, and depression.

Model	Non-Standardized Coefficients		Standardized Coefficients	*t*	Significance
	*B*	Standard Error	Beta		
A. Respondents do not participate in e-learning-based computer programming courses					
(Constant)	2.072	0.466		4.443	0.000
Individual attitude and expectation	0.203	0.102	0.184	1.982	0.049
Challenging goals	−0.034	0.064	−0.043	−0.539	0.590
Clear direction	−0.028	0.104	−0.024	−0.272	0.786
Reward and recognition	−0.068	0.088	−0.065	−0.777	0.438
Punishment	−0.030	0.047	−0.045	−0.623	0.534
Social pressure and competition	0.011	0.054	0.015	0.200	0.842
Anxiety (GAD-7)	0.061	0.011	0.360	5.557	0.000
Depression (PHQ-9)	0.033	0.010	0.219	3.296	0.001
*R* = 0.472; *R* Square = 0.223; Adjusted *R* Square = 0.191; Standard Error of the Estimate = 0.80231; *F* (8, 197) = 7.058, *p* < 0.001					
B. Respondents participate in e-learning-based computer programming courses					
(Constant)	1.712	0.394		4.346	0.000
Individual attitude and expectation	−0.262	0.075	−0.246	−3.514	0.001
Challenging goals	−0.016	0.054	−0.017	−0.298	0.766
Clear direction	0.209	0.088	0.172	2.369	0.019
Reward and recognition	0.121	0.062	0.116	1.957	0.052
Punishment	0.008	0.041	0.011	0.199	0.842
Social pressure and competition	−0.017	0.045	−0.020	−0.374	0.709
Anxiety (GAD-7)	0.039	0.013	0.222	2.907	0.004
Depression (PHQ-9)	0.082	0.012	0.551	6.812	0.000
*R* = 0.768; *R* Square = 0.589; Adjusted *R* Square = 0.571; Standard Error of the Estimate = 0.63359; *F* (8, 176) = 31.554, *p* < 0.001					

**Table 14:** Multiple regression models in which the dependent variable is mental fatigue and the predictors are learning motivating factors, anxiety, and depression.

Model	Non-Standardized Coefficients		Standardized Coefficients	*t*	Significance
	*B*	Standard Error	Beta		
A. Respondents do not participate in e-learning-based computer programming courses					
(Constant)	3.247	0.478		6.787	0.000
Individual attitude and expectation	0.079	0.105	0.072	0.754	0.452
Challenging goals	−0.123	0.066	−0.154	−1.871	0.063
Clear direction	−0.034	0.107	−0.029	−0.320	0.749
Reward and recognition	−0.127	0.090	−0.121	−1.413	0.159
Punishment	0.005	0.049	0.007	0.098	0.922
Social pressure and competition	0.000	0.055	0.001	0.009	0.993
Anxiety (GAD-7)	0.041	0.011	0.243	3.645	0.000
Depression (PHQ-9)	0.026	0.010	0.177	2.580	0.011
*R* = 0.419; *R* Square = 0.175; Adjusted *R* Square = 0.142; Standard Error of the Estimate = 0.82302; *F* (8, 197) = 5.229, *p* < 0.001					
B. Respondents participate in e-learning-based computer programming courses					
(Constant)	2.451	0.416		5.893	0.000
Individual attitude and expectation	−0.131	0.079	−0.123	−1.657	0.099
Challenging goals	−0.252	0.057	−0.274	−4.387	0.000
Clear direction	0.148	0.093	0.122	1.584	0.115
Reward and recognition	0.122	0.065	0.118	1.876	0.062
Punishment	−0.095	0.043	−0.127	−2.217	0.028
Social pressure and competition	0.092	0.047	0.111	1.933	0.055
Anxiety (GAD-7)	0.017	0.014	0.100	1.229	0.221
Depression (PHQ-9)	0.073	0.013	0.490	5.695	0.000
*R* = 0.732; *R* Square = 0.535; Adjusted *R* Square = 0.514; Standard Error of the Estimate = 0.66896; *F* (8, 176) = 25.338, *p* < 0.001					

**Table 15:** Scalar estimates of the model of associations between learning motivating factors, anxiety, depression, and fatigue in the sample of participants of e-learning-based computer programming courses.

Factors		Variables	*B*	*SE*	CR	*β*
Punishment	->	LMF Item 14	1.000			0.929
Punishment	->	LMF Item 15	0.778	0.162	4.804	0.716
Social pressure and competition	->	LMF Item 16	1.000			0.625
Social pressure and competition	->	LMF Item 17	0.877	0.126	6.978	0.636
Social pressure and competition	->	LMF Item 18	1.056	0.134	7.867	0.756
Social pressure and competition	->	LMF Item 19	1.246	0.152	8.183	0.835
Anxiety	->	GAD-7 Item 1	1.000			0.866
Anxiety	->	GAD-7 Item 2	1.027	0.063	16.239	0.874
Anxiety	->	GAD-7 Item 3	1.074	0.067	16.142	0.872
Anxiety	->	GAD-7 Item 4	0.870	0.068	12.832	0.764
Anxiety	->	GAD-7 Item 5	0.701	0.062	11.328	0.704
Anxiety	->	GAD-7 Item 6	0.761	0.065	11.764	0.722
Anxiety	->	GAD-7 Item 7	0.841	0.071	11.795	0.724
Depression	->	PHQ-9 Item 1	1.000			0.746
Depression	->	PHQ-9 Item 2	1.114	0.097	11.511	0.822
Depression	->	PHQ-9 Item 3	1.027	0.108	9.482	0.690
Depression	->	PHQ-9 Item 4	1.028	0.092	11.123	0.797
Depression	->	PHQ-9 Item 5	1.076	0.110	9.780	0.709
Depression	->	PHQ-9 Item 6	1.256	0.111	11.296	0.808
Depression	->	PHQ-9 Item 7	0.838	0.094	8.866	0.648
Depression	->	PHQ-9 Item 8	0.563	0.079	7.134	0.529
Depression	->	PHQ-9 Item 9	0.708	0.080	8.874	0.649
Challenging goals	->	LMF Item 7	1.000			0.794
Challenging goals	->	LMF Item 6	1.141	0.105	10.846	0.874
Challenging goals	->	LMF Item 5	0.798	0.086	9.280	0.688
Anxiety	->	General fatigue	0.081	0.120	0.677	0.071
Depression	->	General fatigue	0.989	0.158	6.254	0.725
Depression	<->	Anxiety	0.506	0.072	7.056	0.831
Depression	<->	Social pressure and competition	−0.146	0.063	−2.313	−0.204
Social pressure and competition	<->	Punishment	0.491	0.126	3.891	0.382
Anxiety	<->	Punishment	−0.091	0.089	−1.016	−0.083
Depression	<->	Challenging goals	−0.339	0.069	−4.906	−0.491
Anxiety	<->	Social pressure and competition	−0.170	0.074	−2.290	−0.199
Depression	<->	Punishment	−0.090	0.075	−1.187	−0.097
Punishment	<->	Challenging goals	0.287	0.109	2.628	0.230
Social pressure and competition	<->	Challenging goals	0.300	0.093	3.217	0.311
Anxiety	<->	Challenging goals	−0.324	0.075	−4.296	−0.393

*SE*, standard error; CR, critical ratio. Group number 1 (Group number 1—Default model); Estimates (Group number 1—Default model); Scalar Estimates (Group number 1—Default model); Maximum Likelihood Estimates; Regression Weights (Group number 1—Default model).

**Table 16:** Scalar estimates of the model of associations between learning motivating factors, anxiety, depression, and fatigue in the sample of respondents who did not participate in e-learning-based computer programming courses.

Factors		Variables	*B*	*SE*	CR	*β*
Punishment	->	LMF Item 14	1.000			0.733
Punishment	->	LMF Item 15	1.065	0.160	6.649	0.828
Social pressure and competition	->	LMF Item 16	1.000			0.733
Social pressure and competition	->	LMF Item 17	0.847	0.090	9.369	0.678
Social pressure and competition	->	LMF Item 18	0.908	0.085	10.637	0.774
Social pressure and competition	->	LMF Item 19	1.115	0.098	11.385	0.847
Anxiety	->	GAD-7 Item 1	1.000			0.794
Anxiety	->	GAD-7 Item 2	1.126	0.073	15.492	0.862
Anxiety	->	GAD-7 Item 3	1.189	0.073	16.209	0.892
Anxiety	->	GAD-7 Item 4	0.995	0.074	13.364	0.771
Anxiety	->	GAD-7 Item 5	0.857	0.071	12.027	0.709
Anxiety	->	GAD-7 Item 6	0.929	0.081	11.478	0.683
Anxiety	->	GAD-7 Item 7	0.995	0.082	12.118	0.714
Depression	->	PHQ-9 Item 1	1.000			0.773
Depression	->	PHQ-9 Item 2	1.098	0.093	11.774	0.791
Depression	->	PHQ-9 Item 3	1.051	0.115	9.110	0.631
Depression	->	PHQ-9 Item 4	1.138	0.099	11.487	0.774
Depression	->	PHQ-9 Item 5	1.047	0.103	10.185	0.697
Depression	->	PHQ-9 Item 6	1.128	0.102	11.039	0.748
Depression	->	PHQ-9 Item 7	0.807	0.096	8.386	0.586
Depression	->	PHQ-9 Item 8	0.550	0.080	6.875	0.487
Depression	->	PHQ-9 Item 9	0.561	0.081	6.911	0.490
Challenging goals	->	LMF Item 7	1.000			0.858
Challenging goals	->	LMF Item 6	1.061	0.065	16.352	0.911
Challenging goals	->	LMF Item 5	0.895	0.061	14.555	0.815
Anxiety	->	General fatigue	0.443	0.081	5.478	0.348
Depression	->	General fatigue	0.335	0.091	3.668	0.251
Depression	<->	Anxiety	0.120	0.038	3.173	0.251
Depression	<->	Social pressure and competition	−0.064	0.063	1.017	-0.081
Social pressure and competition	<->	Punishment	0.739	0.149	4.951	0.555
Anxiety	<->	Punishment	−0.008	0.064	−0.124	−0.010
Depression	<->	Challenging goals	−0.214	0.060	−3.588	−0.294
Anxiety	<->	Social pressure and competition	−0.004	0.063	−0.068	−0.005
Depression	<->	Punishment	−0.038	0.064	−0.593	−0.050
Punishment	<->	Challenging goals	0.268	0.105	2.558	0.219
Social pressure and competition	<->	Challenging goals	0.424	0.106	3.982	0.334
Anxiety	<->	Challenging goals	−0.128	0.057	−2.230	−0.168

**Table 17:** Results of structural equation modeling of challenging goals, mental fatigue, depression, and anxiety in the group of participants of e-learning-based computer programming courses.

Factors		Factors	*B*	*SE*	*z*	*p*	LL	UL	*β*	*R^2^*
Challenging goals	->	Mental fatigue	−0.467	0.061	−7.717	<0.001	−0.586	−0.349	−0.507	0.257
Mental fatigue	->	Depression	4.471	0.345	12.967	<0.001	3.795	5.147	0.664	0.441
Depression	->	Anxiety	0.659	0.040	16.631	<0.001	0.582	0.737	0.769	0.591

LL, lower limit; UL, upper limit.

**Table 18:** Results of structural equation modelling of challenging goals, mental fatigue, depression, and anxiety in groups of participants and non-participants of e-learning-based computer programming courses.

Factors		Factors	*B*	*SE*	*z*	*p*	LL	UL	*β*	Group *
Challenging goals	->	Mental fatigue	−0.467	0.061	−7.717	<0.001	−0.586	−0.349	−0.507	2
Mental fatigue	->	Depression	4.471	0.345	12.967	<0.001	3.795	5.147	0.664	2
Mental fatigue	->	Anxiety	3.262	0.335	9.726	<0.001	2.605	3.920	0.565	2
Challenging goals	->	Mental fatigue	−0.187	0.057	−3.298	<0.001	−0.298	−0.076	−0.235	1
Mental fatigue	->	Depression	1.871	0.441	4.239	<0.001	1.006	2.736	0.277	1
Mental fatigue	->	Anxiety	1.833	0.425	4.313	<0.001	1.000	2.665	0.309	1

* Group 2: participants of e-learning-based computer programming courses; Group 1: non-participants of e-learning-based computer programming courses.

## Data Availability

The data that support the findings of this study are available from the corresponding author, upon reasonable request.

## References

[1] Blut M., Wang C. (2020). Technology readiness: A meta-analysis of conceptualizations of the construct and its impact on technology usage. J. Acad. Mark. Sci..

[2] Scherer R., Siddiq F., Sánchez Viveros B. (2019). The cognitive benefits of learning computer programming: A meta-analysis of transfer effects. J. Educ. Psychol..

[3] Brehm L., Günzel H. Learning Lab “Digital Technologies”—Concept, Streams and Experiences. Proceedings of the 4th International Conference on Higher Education Advances.

[4] HackerRank Developer Skills Report (2021). Insights Based on 116,648 Developers. www.hackerrank.com.

[5] 11 Most In-Demand Programming Languages in 2021. https://bootcamp.berkeley.edu/blog/most-in-demand-programming-languages/.

[6] Law K.M.Y., Lee V.C.S., Yu Y.T. (2010). Learning motivation in e-learning facilitated computer programming courses. Comput. Educ..

[7] Hawi N. (2010). Causal attributions of success and failure made by undergraduate students in an introductory-level computer programming course. Comput. Educ..

[8] Govender I. (2009). The learning context: Influence on learning to program. Comput. Educ..

[9] Serrano-Cámara L.M., Paredes-Velasco M., Alcover C.-M., Velazquez-Iturbide J.Á. (2014). An evaluation of students’ motivation in computer-supported collaborative learning of programming concepts. Comput. Hum. Behav..

[10] Kintu M.J., Zhu C., Kagambe E. (2017). Blended learning effectiveness: The relationship between student characteristics, design features and outcomes. Int. J. Educ. Technol. High. Educ..

[11] Law K.M.Y., Geng S. (2019). How innovativeness and handedness affect learning performance of engineering students?. Int. J. Technol. Des. Educ..

[12] Law K.M.Y., Geng S., Li T. (2019). Student enrollment, motivation and learning performance in a blended learning environment: The mediating effects of social, teaching, and cognitive presence. Comput. Educ..

[13] Ryan R.M., Deci E.L. (2000). Self-determination theory and the facilitation of intrinsic motivation, social development, and well-being. Am. Psychol..

[14] Law K.M.Y., Breznik K. (2017). Impacts of innovativeness and attitude on entrepreneurial intention: Among engineering and non-engineering students. Int. J. Technol. Des. Educ..

[15] Zimmerman B.J., Schunk D.H. (2008). Motivation: An essential dimension of self-regulated learning. Motivation and Self-Regulated Learning: Theory, Research, and Applications.

[16] Long M., Wood C., Littleton K., Passenger T., Sheehy K. (2010). The Psychology of Education.

[17] Vroom V.H. (1964). Work and Motivation.

[18] Harackiewicz J.M., Barron K.E., Carter S.M., Lehto A.T., Elliot A.J. (1997). Predictors and consequences of achievement goals in the college classroom: Maintaining interest and making the grade. J. Pers. Soc. Psychol..

[19] Harackiewicz J.M., Barron K.E., Elliot A.J. (1998). Rethinking achievement goals: When are they adaptive for college students and why?. Educ. Psychol..

[20] Harackiewicz J.M., Barron K.E., Tauer J.M., Elliot A.J. (2002). Predicting success in college: A longitudinal study of achievement goals and ability measures as predictors of interest and performance from freshman year through graduation. J. Educ. Psychol..

[21] Hendry G.D., Lyon P.M., Prosser M., Sze D. (2006). Conceptions of problem-based learning: The perspectives of students entering a problem-based medical program. Med. Teach..

[22] Stipek D.J. (1996). Motivation and instruction. Handbook of Educational Psychology.

[23] Jenkins T. (2001). The motivation of students of programming. Proceedings of the 6th annual conference on Innovation and technology in computer science education (ITiCSE ’01).

[24] Skinner B.F. Contingencies of Reinforcement: A Theoretical Analysis.

[25] Chan C.C.A., Pearson C., Entrekin L. (2003). Examining the effects of internal and external team learning on team performance. Team Perform. Manag. Int. J..

[26] Rassuli A., Manzer J.P. (2005). “Teach Us to Learn”: Multivariate Analysis of Perception of Success in Team Learning. J. Educ. Bus..

[27] Lee Y., Ertmer P.A. (2006). Examining the Effect of Small Group Discussions and Question Prompts on Vicarious Learning Outcomes. J. Res. Technol. Educ..

[28] Zimmerman B.J., Kitsantas A. (2005). The Hidden Dimension of Personal Competence: Self-Regulated Learning and Practice. Handbook of Competence and Motivation.

[29] Locke E.A., Latham G.P. (1990). A Theory of Goal Setting & Task Performance.

[30] Bong M. (2004). Academic Motivation in Self-Efficacy, Task Value, Achievement Goal Orientations, and Attributional Beliefs. J. Educ. Res..

[31] Margolis H., McCabe P.P. (2004). Self-Efficacy: A Key to Improving the Motivation of Struggling Learners. Clear. House.

[32] Barak M. (2010). Motivating self-regulated learning in technology education. Int. J. Technol. Des. Educ..

[33] Ng C. (2021). What kind of students persist in science learning in the face of academic challenges?. J. Res. Sci. Teach..

[34] Zatarain Cabada R., Barrón Estrada M.L., Ríos Félix J.M., Alor Hernández G. (2020). A virtual environment for learning computer coding using gamification and emotion recognition. Interact. Learn. Environ..

[35] Lin Y.G., McKeachie W.J., Kim Y.C. (2003). College student intrinsic and/or extrinsic motivation and learning. Learn. Individ. Differ..

[36] Yin J., Goh T.-T., Yang B., Xiaobin Y. (2021). Conversation Technology With Micro-Learning: The Impact of Chatbot-Based Learning on Students’ Learning Motivation and Performance. J. Educ. Comput. Res..

[37] Bosch N., D’Mello S., Mills C. What Emotions Do Novices Experience during Their First Computer Programming Learning Session?. Proceedings of the International Conference on Artificial Intelligence in Education.

[38] Bosch N., Chen Y., D’Mello S. It’s Written on Your Face: Detecting Affective States from Facial Expressions while Learning Computer Programming. Proceedings of the International Conference on Intelligent Tutoring Systems.

[39] Bosch N., D’Mello S. (2017). The Affective Experience of Novice Computer Programmers. Int. J. Artif. Intell. Educ..

[40] Lee D.M.C., Rodrigo M.M.T., Baker R.S.J.D., Sugay J.O., Coronel A. (2011). Exploring the relationship between novice programmer confusion and achievement. Lecture Notes in Computer Science (Including Subseries Lecture Notes in Artificial Intelligence and Lecture Notes in Bioinformatics.

[41] Bosch N., D’Mello S., Baker R., Ocumpaugh J., Shute V., Ventura M., Wang L., Zhao W. (2015). Automatic Detection of Learning-Centered Affective States in the Wild. Proceedings of the 20th International Conference on Intelligent User Interfaces.

[42] Bahreini K., Nadolski R., Westera W. (2016). Towards multimodal emotion recognition in e-learning environments. Interact. Learn. Environ..

[43] Lin H.C.K., Su S.H., Chao C.J., Hsieh C.Y., Tsai S.C. (2016). Construction of multi-mode affective learning system: Taking Affective Design as an Example. Educ. Technol. Soc..

[44] D’mello S.K., Kory J. (2015). A Review and Meta-Analysis of Multimodal Affect Detection Systems. ACM Comput. Surv..

[45] Kim Y., Lee H., Provost E.M. Deep learning for robust feature generation in audiovisual emotion recognition. Proceedings of the 2013 IEEE International Conference on Acoustics, Speech and Signal Processing.

[46] Ninaus M., Moeller K., McMullen J., Kiili K. (2017). Acceptance of Game-Based Learning and Intrinsic Motivation as Predictors for Learning Success and Flow Experience. Int. J. Serious Games.

[47] Hong J.-C., Hwang M.-Y., Tai K.-H., Lin P.-H. (2017). Intrinsic motivation of Chinese learning in predicting online learning self-efficacy and flow experience relevant to students’ learning progress. Comput. Assist. Lang. Learn..

[48] Luo Z., Subramaniam G., Steen B. (2020). Will anxiety boost motivation? The relationship between anxiety and motivation in foreign language learning. Malays. J. ELT Res..

[49] Xiu Y., Thompson P. (2020). Flipped University Class: A Study of Motivation and Learning. J. Inf. Technol. Educ. Res..

[50] Young A.M., Wendel P.J., Esson J.M., Plank K.M. (2018). Motivational decline and recovery in higher education STEM courses. Int. J. Sci. Educ..

[51] Hohoff C. (2009). Anxiety in mice and men: A comparison. J. Neural Transm..

[52] Gordon J.A., Hen R. (2004). Genetic Approaches to the Study of Anxiety. Annu. Rev. Neurosci..

[53] Gatt J.M., Nemeroff C.B., Dobson-Stone C., Paul R.H., Bryant R.A., Schofield P.R., Gordon E., Kemp A.H., Williams L.M. (2009). Interactions between BDNF Val66Met polymorphism and early life stress predict brain and arousal pathways to syndromal depression and anxiety. Mol. Psychiatry.

[54] Brown R.J., Skelly N., Chew-Graham C.A. (2020). Online health research and health anxiety: A systematic review and conceptual integration. Clin. Psychol. Sci. Pract..

[55] Garfin D.R., Silver R.C., Holman E.A. (2020). The novel coronavirus (COVID-2019) outbreak: Amplification of public health consequences by media exposure. Health Psychol..

[56] Jungmann S.M., Witthöft M. (2020). Health anxiety, cyberchondria, and coping in the current COVID-19 pandemic: Which factors are related to coronavirus anxiety?. J. Anxiety Disord..

[57] Clark L.A., Watson D. (1991). Tripartite model of anxiety and depression: Psychometric evidence and taxonomic implications. J. Abnorm. Psychol..

[58] Renner K.H., Hock M., Bergner-Köther R., Laux L. (2018). Differentiating anxiety and depression: The State-Trait Anxiety-Depression Inventory. Cogn. Emot..

[59] Iversen A., Wessely S. (2003). Chronic fatigue and depression. Curr. Opin. Psychiatry.

[60] Payne S.C., Youngcourt S.S., Beaubien J.M. (2007). A meta-analytic examination of the goal orientation nomological net. J. Appl. Psychol..

[61] Vedel A. (2014). The Big Five and tertiary academic performance: A systematic review and meta-analysis. Pers. Individ. Dif..

[62] Ruiz J.G., Mintzer M.J., Leipzig R.M. (2006). The Impact of E-Learning in Medical Education. Acad. Med..

[63] Sun P.-C., Tsai R.J., Finger G., Chen Y.-Y., Yeh D. (2008). What drives a successful e-Learning? An empirical investigation of the critical factors influencing learner satisfaction. Comput. Educ..

[64] Lee M.C. (2010). Explaining and predicting users’ continuance intention toward e-learning: An extension of the expectation-confirmation model. Comput. Educ..

[65] Lawrence S.A., Garcia J., Stewart C., Rodriguez C. (2021). The mental and behavioral health impact of COVID-19 stay at home orders on social work students. Soc. Work Educ..

[66] Escudero-Castillo I., Mato-Díaz F.J., Rodriguez-Alvarez A. (2021). Furloughs, Teleworking and Other Work Situations during the COVID-19 Lockdown: Impact on Mental Well-Being. Int. J. Environ. Res. Public Health.

[67] Fawaz M., Samaha A. (2021). e-learning: Depression, anxiety, and stress symptomatology among Lebanese university students during COVID-19 quarantine. Nurs. Forum.

[68] Othman N., Ahmad F., El Morr C., Ritvo P. (2019). Perceived impact of contextual determinants on depression, anxiety and stress: A survey with university students. Int. J. Ment. Health Syst..

[69] Nurunnabi M., Hossain S.F.A.H., Chinna K., Sundarasen S., Khoshaim H.B., Kamaludin K., Baloch G.M., Sukayt A., Shan X. (2020). Coping strategies of students for anxiety during the COVID-19 pandemic in China: A cross-sectional study. F1000Research.

[70] Jojoa M., Lazaro E., Garcia-Zapirain B., Gonzalez M.J., Urizar E. (2021). The Impact of COVID 19 on University Staff and Students from Iberoamerica: Online Learning and Teaching Experience. Int. J. Environ. Res. Public Health.

[71] Brooks S.K., Webster R.K., Smith L.E., Woodland L., Wessely S., Greenberg N., Rubin G.J. (2020). The psychological impact of quarantine and how to reduce it: Rapid review of the evidence. Lancet.

[72] Odriozola-González P., Planchuelo-Gómez Á., Irurtia M.J., de Luis-García R. (2020). Psychological effects of the COVID-19 outbreak and lockdown among students and workers of a Spanish university. Psychiatry Res..

[73] Cao W., Fang Z., Hou G., Han M., Xu X., Dong J., Zheng J. (2020). The psychological impact of the COVID-19 epidemic on college students in China. Psychiatry Res..

[74] Aslan I., Ochnik D., Çınar O. (2020). Exploring Perceived Stress among Students in Turkey during the COVID-19 Pandemic. Int. J. Environ. Res. Public Health.

[75] MacWhinnie S.G.B., Mitchell C. (2017). English classroom reforms in Japan: A study of Japanese university EFL student anxiety and motivation. Asian-Pac. J. Second Foreign Lang. Educ..

[76] Al-Tammemi A.B., Akour A., Alfalah L. (2020). Is It Just About Physical Health? An Online Cross-Sectional Study Exploring the Psychological Distress Among University Students in Jordan in the Midst of COVID-19 Pandemic. Front. Psychol..

[77] Friedman N.P., Miyake A., Young S.E., DeFries J.C., Corley R.P., Hewitt J.K. (2008). Individual differences in executive functions are almost entirely genetic in origin. J. Exp. Psychol. Gen..

[78] Hong J.-C., Hwang M.-Y., Chang H.-W., Tai K.-H., Kuo Y.-C., Tsai Y.-H. (2015). Internet cognitive failure and fatigue relevant to learners’ self-regulation and learning progress in English vocabulary with a calibration scheme. J. Comput. Assist. Learn..

[79] Christodoulou C. (2005). The Assessment and Measurement of Fatigue. Fatigue as A Window to the Brain.

[80] DeLuca J. (2005). Fatigue, Cognition, and Mental Effort. Fatigue as A Window to the Brain.

[81] Kanfer R. (2011). Determinants and consequences of subjective cognitive fatigue. Cognitive Fatigue: Multidisciplinary Perspectives on Current Research and Future Applications.

[82] Ding L., Velicer W.F., Harlow L.L. (1995). Effects of estimation methods, number of indicators per factor, and improper solutions on structural equation modeling fit indices. Struct. Equ. Model. Multidiscip. J..

[83] Law K.M.Y., Sandnes F.E., Jian H.L., Huang Y.P. (2009). A comparative study of learning motivation among engineering students in south east asia and beyond. Int. J. Eng. Educ..

[84] Kroenke K., Spitzer R.L., Williams J.B.W. (2001). The PHQ-9. J. Gen. Intern. Med..

[85] Spitzer R.L., Kroenke K., Williams J.B.W., Löwe B. (2006). A Brief Measure for Assessing Generalized Anxiety Disorder. Arch. Intern. Med..

[86] Smets E.M.A., Garssen B., Bonke B., De Haes J.C.J.M. (1995). The multidimensional Fatigue Inventory (MFI) psychometric qualities of an instrument to assess fatigue. J. Psychosom. Res..

[87] Kocalevent R.-D., Hinz A., Brähler E. (2013). Standardization of the depression screener Patient Health Questionnaire (PHQ-9) in the general population. Gen. Hosp. Psychiatry.

[88] Löwe B., Decker O., Müller S., Brähler E., Schellberg D., Herzog W., Herzberg P.Y. (2008). Validation and Standardization of the Generalized Anxiety Disorder Screener (GAD-7) in the General Population. Med. Care.

[89] Schwarz R., Krauss O., Hinz A. (2003). Fatigue in the General Population. Oncol. Res. Treat..

[90] Kline R.B. (2016). Principles and Practice of Structural Equation Modeling.

[91] Bryne B.M. (2013). Structural Equation Modeling with AMOS: Basic Concepts, Applications, and Programming.

[92] Murtaugh P.A. (2014). In defense of P values. Ecology.

[93] Tang Y.M., Chen P.C., Law K.M.Y., Wu C.H., Lau Y., Guan J., He D., Ho G.T.S. (2021). Comparative analysis of Student’s live online learning readiness during the coronavirus (COVID-19) pandemic in the higher education sector. Comput. Educ..

[94] Frey A.-L., McCabe C. (2020). Impaired social learning predicts reduced real-life motivation in individuals with depression: A computational fMRI study. J. Affect. Disord..

[95] McRae K., Gross J.J. (2020). Emotion regulation. Emotion.

[96] Brackney B.E., Karabenick S.A. (1995). Psychopathology and academic performance: The role of motivation and learning strategies. J. Couns. Psychol..

[97] Garvik M., Idsoe T., Bru E. (2016). Motivation and Social Relations in School Following a CBT Course for Adolescents With Depressive Symptoms: An Effectiveness Study. Scand. J. Educ. Res..

[98] Au R.C.P., Watkins D., Hattie J., Alexander P. (2009). Reformulating the depression model of learned hopelessness for academic outcomes. Educ. Res. Rev..

[99] Roeser R.W., Strobel K.R., Quihuis G. (2002). Studying Early Adolescents’ Academic Motivation, Social-Emotional Functioning, and Engagement in Learning: Variable- and Person-Centered Approaches. Anxiety Stress Coping.

[100] Essau C.A., Leung P.W.L., Conradt J., Cheng H., Wong T. (2008). Anxiety symptoms in Chinese and German adolescents: Their relationship with early learning experiences, perfectionism, and learning motivation. Depress. Anxiety.

[101] Zeng L., Chen D., Xiong K., Pang A., Huang J., Zeng L. Medical University Students’ Personality and Learning Performance: Learning Burnout as a Mediator. Proceedings of the 2015 7th International Conference on Information Technology in Medicine and Education.

[102] Lin S.-H., Huang Y.-C. (2012). Investigating the relationships between loneliness and learning burnout. Act. Learn. High. Educ..

[103] van Vendeloo S.N., Prins D.J., Verheyen C.C.P.M., Prins J.T., van den Heijkant F., van der Heijden F.M.M.A., Brand P.L.P. (2018). The learning environment and resident burnout: A national study. Perspect. Med. Educ..

[104] Zhang J.-Y., Shu T., Xiang M., Feng Z.-C. (2021). Learning Burnout: Evaluating the Role of Social Support in Medical Students. Front. Psychol..

[105] Lenaert B., Boddez Y., Vlaeyen J.W.S., van Heugten C.M. (2018). Learning to feel tired: A learning trajectory towards chronic fatigue. Behav. Res. Ther..

[106] Asadayoobi N., Jaber M.Y., Taghipour S. (2021). A new learning curve with fatigue-dependent learning rate. Appl. Math. Model..

[107] Forsythe A., Jellicoe M. (2018). Predicting gainful learning in Higher Education: A goal-orientation approach. High. Educ. Pedagog..

[108] Mills J.S., Blankstein K.R. (2000). Perfectionism, intrinsic vs extrinsic motivation, and motivated strategies for learning: A multidimensional analysis of university students. Pers. Individ. Dif..

[109] Nguyen T.D., Shultz C.J., Westbrook M.D. (2012). Psychological Hardiness in Learning and Quality of College Life of Business Students: Evidence from Vietnam. J. Happiness Stud..

[110] Li W., Lee A.M., Solmon M. (2008). Effects of Dispositional Ability Conceptions, Manipulated Learning Environments, and Intrinsic Motivation on Persistence and Performance. Res. Q. Exerc. Sport.

[111] Karlen Y., Suter F., Hirt C., Maag Merki K. (2019). The role of implicit theories in students’ grit, achievement goals, intrinsic and extrinsic motivation, and achievement in the context of a long-term challenging task. Learn. Individ. Differ..

[112] Rigolizzo M., Zhu Z. (2021). The ebb and flow of learning motivation: The differentiated impact of the implicit theory of intelligence on learning behaviors. Hum. Resour. Dev. Q..

[113] Reis S., Coelho F., Coelho L. (2020). Success Factors in Students’ Motivation with Project Based Learning: From Theory to Reality. Int. J. Online Biomed. Eng..

[114] Truzoli R., Viganò C., Galmozzi P.G., Reed P. (2020). Problematic internet use and study motivation in higher education. J. Comput. Assist. Learn..

[115] Reed P., Reay E. (2015). Relationship between levels of problematic Internet usage and motivation to study in university students. High. Educ..

[116] Santamaría-Vázquez M., Del Líbano M., Martínez-Lezaun I., Ortiz-Huerta J.H. (2021). Self-Regulation of Motivation and Confinement by COVID-19: A Study in Spanish University Students. Sustainability.

[117] Moore K.P., Richards A.S. (2019). The Effects of Instructor Credibility, Grade Incentives, and Framing of a Technology Policy on Students’ Intent to Comply and Motivation to Learn. Commun. Stud..

[118] Murty V.P., LaBar K.S., Hamilton D.A., Adcock R.A. (2011). Is all motivation good for learning? Dissociable influences of approach and avoidance motivation in declarative memory. Learn. Mem..

[119] Luria E., Shalom M., Levy D.A. (2021). Cognitive Neuroscience Perspectives on Motivation and Learning: Revisiting Self-Determination Theory. Mind Brain Educ..

[120] Jayalath J., Esichaikul V. (2020). Gamification to Enhance Motivation and Engagement in Blended eLearning for Technical and Vocational Education and Training. Technol. Knowl. Learn..

[121] Borovay L.A., Shore B.M., Caccese C., Yang E., Hua O. (2019). Flow, Achievement Level, and Inquiry-Based Learning. J. Adv. Acad..

